# Introgression of a synthetic sex ratio distortion transgene into different genetic backgrounds of *Anopheles coluzzii*

**DOI:** 10.1111/imb.12813

**Published:** 2022-10-31

**Authors:** Paola Pollegioni, Tania Persampieri, Roxana L. Minuz, Alessandro Bucci, Alessandro Trusso, Salvatore Di Martino, Chiara Leo, Marco Bruttini, Marco Ciolfi, Ann‐Marie Waldvogel, Frédéric Tripet, Alekos Simoni, Andrea Crisanti, Ruth Müller

**Affiliations:** ^1^ Research Institute on Terrestrial Ecosystems National Research Council Terni Italy; ^2^ Genetics and Ecology Research Centre Polo d'Innovazione di Genomica, Genetica e Biologia Terni Italy; ^3^ Tuscan Centre of Precision Medicine, Department of Medicine, Surgery and Neurosciences University of Siena Siena Italy; ^4^ Institute of Zoology University of Cologne Köln Germany; ^5^ Centre for Applied Entomology and Parasitology Keele University Newcastle‐under‐Lyme UK; ^6^ Department of Molecular Medicine University of Padova Padova Italy; ^7^ Unit Entomology, Department of Biomedical Sciences Institute of Tropical Medicine Antwerp Belgium

**Keywords:** anopheles, genomic divergence, introgression, malaria vector, sex‐ratio distorter

## Abstract

The development of genetically modified mosquitoes (GMM) and their subsequent field release offers innovative approaches for vector control of malaria. A non‐gene drive self‐limiting male‐bias Ag(PMB)1 strain has been developed in a 47‐year‐old laboratory G3 strain of *Anopheles gambiae s.l*. When Ag(PMB)1 males are crossed to wild‐type females, expression of the endonuclease I‐PpoI during spermatogenesis causes the meiotic cleavage of the X chromosome in sperm cells, leading to fertile offspring with a 95% male bias. However, World Health Organization states that the functionality of the transgene could differ when inserted in different genetic backgrounds of *Anopheles coluzzii* which is currently a predominant species in several West‐African countries and thus a likely recipient for a potential release of self‐limiting GMMs. In this study, we introgressed the transgene from the donor Ag(PMB)1 by six serial backcrosses into two recipient colonies of *An. coluzzii* that had been isolated in Mali and Burkina Faso. Scans of informative Single Nucleotide Polymorphism (SNP) markers and whole‐genome sequencing analysis revealed a nearly complete introgression of chromosomes 3 and X, but a remarkable genomic divergence in a large region of chromosome 2 between the later backcrossed (BC6) transgenic offspring and the recipient paternal strains. These findings suggested to extend the backcrossing breeding strategy beyond BC6 generation and increasing the introgression efficiency of critical regions that have ecological and epidemiological implications through the targeted selection of specific markers. Disregarding differential introgression efficiency, we concluded that the phenotype of the sex ratio distorter is stable in the BC6 introgressed *An. coluzzii* strains.

## Graphical abstract

The introgression of a synthetic sex ratio distortion transgene into two different genetic backgrounds of Anopheles coluzzii by six backcrosses. We showed that the phenotype of the sex ratio distorter is stabile in the introgressed An. coluzzii strains. Complete introgression of chromosome 3 and X, but a remarkable genomic divergence in the chromosome 2 was observed.

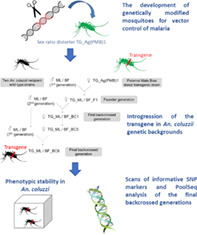

## INTRODUCTION

Malaria still persists as the preeminent arthropod‐borne disease, imposing around half a million deaths every year, even if overall mortality rates have been significantly reduced in the last two decades due to increased prevention and control measures against the *Plasmodium* transmitting *Anopheles* mosquitoes (WHO, 2018). Although conventional insecticides and insecticide‐treated bed nets remain the primary public health strategies, concerns about widespread increased mosquito insecticide resistance and atypical biting behaviour of *Anopheles* mosquitoes (Sougoufara et al., 2017) led to the development of complementary vector control tools including the use of genetically modified mosquitoes (GMM) and their subsequent field release for population control (Burt, 2014). Many different genetic modifications have been proposed and classified according to their ability to spread through natural target populations with a Mendelian (self‐limiting non‐driving strategies) or biased‐Mendelian inheritance (self‐sustaining strategies) such as ‘gene‐drive’ systems (Alphey et al., 2020).

One of the strategies being actively developed aims to suppress the population of vector mosquitoes by spreading a transgenic construct that blocks female reproduction or significantly biases the sex ratio toward males (Hammond & Galizi, 2017). The autosomal sex ratio distorters have been generated in *Anopheles gambiae s.l*. mosquitoes by using site‐specific endonucleases such as I‐PpoI (Galizi et al., 2014; Haghighat‐Khah et al., 2020; Pollegioni et al., 2020). This enzyme promotes the highly specific cleavage of a short‐conserved sequence within the 28 S rDNA repeats located on the X chromosome in the *An. gambiae* complex. In particular, the Paternal Male Bias transgenic line Ag(PMB)1 [formerly called gfp124L‐2] (Galizi et al., 2014) contains the construct [3xP3‐DsRed]β2‐eGFP::I‐PpoI‐124L that encodes a variant of the I‐PpoI endonuclease (mutation W124L) fused to the enhanced Green Fluorescent Protein (eGFP) under the control of the beta‐2 tubulin promoter, which is active during male meiosis. When Ag(PMB)1 males are crossed to wild‐type females, the expression of I‐PpoI endonuclease causes the meiotic cleavage of X‐linked rDNA repeats, affecting the X‐bearing sperm to fertilize the female oocyte, leading to fertile offspring with a 95% male bias and a 50% transgene inheritance. Although a single copy integration of the [3xP3‐DsRed]β2‐eGFP::I‐PpoI‐124L transgene has been initially attested in the chromosome band 3R‐36D for Ag(PMB)1 strain (Galizi et al., 2014), this integration site has been recently revised and re‐assigned to a poorly annotated centromeric region of chromosome 2R 19D (Vitale et al., 2022). Transgenic lines are typically developed in laboratory strains, and in the case of Ag(PMB)1 developed in the G3 wildtype colony of *Anopheles* mosquitoes, which was isolated in The Gambia (Africa) and has been maintained in the laboratory for approximately 47 years (Malaria Research and Reference Reagent Resource Center, Atlanta, USA; stock number MRA‐112). Although the G3 strain has been classified as *An. gambiae* sensu *strictu* for decades, it is now supposed to be a mongrel stock between *An. gambiae* and *Anopheles coluzzii* (https://www.beiresources.org/Catalog/BEIVectors/MRA-112.aspx).

In line with World Health Organization guidance on the development and testing of GM mosquito technologies (WHO/TDR & FNIH, 2021), the dynamics of the [3xP3‐DsRed]β2‐eGFP::I‐PpoI‐124L transgene carried by Ag(PMB)1 strain have been successfully assessed in indoor large cages, testing the potential of GM mosquitoes to suppress vector populations if released in large numbers (Facchinelli et al., 2019) and providing a demonstration of the self‐limiting nature of the transgene (Pollegioni et al., 2020). The phased approach suggested by World Health Organization includes the evaluation of phenotypic stability of the transgene (e.g., male bias, female fertility) in a variety of genetic backgrounds through indoor‐cage studies. *An. coluzzii* is currently a predominant species in several West‐African countries and thus a likely recipient of a potential release of self‐limiting GMMs. Therefore, we monitored the phenotypic parameters of the transgene in two distinct *An. coluzzii* genetic backgrounds.

*Anopheles gambiae* and *An. coluzzii*, are two major African malaria vectors, morphologically indistinguishable but characterized by a widespread genomic divergence, highly pronounced at pericentromeric regions of each chromosome (islands of divergence) (Neafsey et al., 2010; White et al., 2010). These sister species differ in their ecological niche partitioning at the larval stage (Tene Fossog et al., 2015) and in swarming behaviour, favouring their assortative mating (Diabaté et al., 2009; Simões et al., 2017). Despite the lack of intrinsic postzygotic isolation in the laboratory (Diabate et al., 2007), it was postulated that these species have been largely reproductively isolated in most places where they occur in sympatry (White et al., 2010). Indeed, low *An. coluzzii* × *An. gambiae* s.s. hybrid rates at the adult stage have been reported in most of the natural populations of West Africa, with a 95% C.I. frequency of 0.18%–0.81%, except for the ‘high hybridization area’ (HHA) at the westernmost limit of their distribution range, including coastal fringe of Guinea Bissau, the estuary of the river Gambia and Casamance in Senegal (Pombi et al., 2017). However, a large body of work revealed that assortative mating may be occasionally disrupted (Lee et al., 2013; Vicente et al., 2017). Hybridization and subsequent adaptive introgression occurred between *An. gambiae* and *An. coluzzii* in West Africa starting in 2006, coincident with an increase in insecticide‐treated bed net usage (Clarkson et al., 2014; Norris et al., 2015). Given the possibility of the inter‐specific gene flow between *An. coluzzii* and *An. gambiae* which would involve a variety of wild‐type genetic backgrounds, an evaluation of functional properties of the transgenes after their introgression into these closely‐related vector species would be of great importance.

**FIGURE 1:**
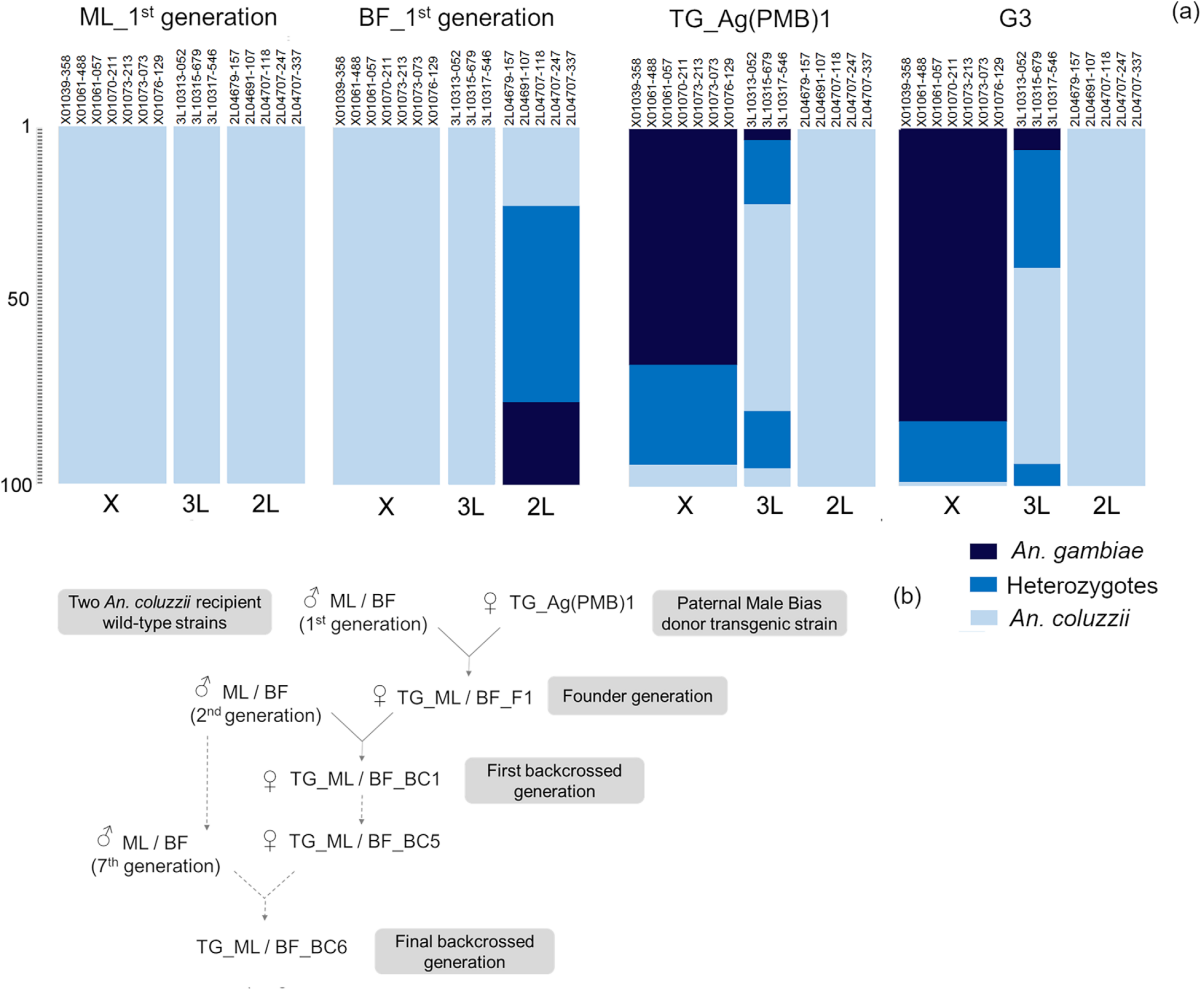
Introgression of the sex ratio distorter from the donor Ag(PMB)1 to the recipient *An. Coluzzii* wild‐type strains, Mali‐NIH (ML) and BF_Ac(WT) (BF). (a) Patterns of genetic admixture between *An. Gambiae* and *An. Coluzzii* for three parental strains and G3 control, using DIS genotyping assay (Lee et al., 2013). First mosquito generation used for starting the introgression process was displayed for each strain, Ag(PMB)1, ML_1st generation and BF_1st generation. Columns represent 15 discriminative SNPs for *An. Coluzzi* and *An. Gambiae* is located in islands of genomic divergence in chromosome X (7 SNPs spanning 4.4 Mbp), 2L (5 SNPs spanning 2.2 Mbp) and 3L (3 SNPs spanning 117 kbp). 100 individual mosquitoes are represented by coloured horizontal lines, with individuals stacked vertically. (b) Schematic diagram of the introgression of [3xP3‐DsRed]β2‐eGFP::I‐PpoI‐124L construct from the donor Ag(PMB)1 transgenic line developed using G3 laboratory strain (Galizi et al., 2014), into two genetic background of *An. Coluzzii* laboratory strains by six serial backcrosses

The aim of this study was to test the efficiency of the introgression of a synthetic sex ratio distortion transgene into different genetic backgrounds of *An. gambiae* (s.l.) sibling species. Therefore, we introgressed the [3xP3‐DsRed]β2‐eGFP::I‐PpoI‐124L transgene from the donor strain Ag(PMB)1 into two recipient wild‐type *An. coluzzii* strains collected in Mali and Burkina Faso before and after 2006, respectively (Figure 1, Table S1). Following the backcrossing breeding approach (Reyes‐Valdés, 2000) and assuming Hardy–Weinberg equilibrium, we hypothesized a 99.22% recovery of the recipient parental genome in each of the new introgressed transgenic strains (TG) after six serial backcrosses (BC6) with recipient wild‐type strains. Our subsequent objectives were (1) understanding the phenotypic stability of the [3xP3‐DsRed]β2‐eGFP::I‐PpoI‐124L sex ratio distorter in two *An. coluzzii* genetic backgrounds from Burkina Faso (BF) and Mali (ML) by monitoring life‐history parameters (2) quantifying and comparing the degree of genomic introgression of *An. coluzzii* from distinct genetic backgrounds into the final backcrossed transgenic strains using the Divergence Island SNP (DIS) Genotyping Assay (Lee et al., 2013), Ancestry Informative Markers analysis (AIMs) (Vicente et al., 2017) and genome‐wide pooled population sequencing (Pool‐Seq) approach (Futschik & Schlötterer, 2010), and (3) highlighting genomic regions in the backcrossed transgenic individuals showing a differential introgression rate and unexpected high genetic differentiation in comparison to recipient wild‐type *An. coluzzii* strains.

## RESULTS

### Sex ratio distortion and life history parameters in the transgenic backcrossed 6 (TG_BC6) offspring

After the introgression of the [3xP3‐DsRed]β2‐eGFP::I‐PpoI‐124L construct from the donor Ag(PMB)1 into the genetic background of two recipients *An. coluzzii* wild‐type colonies, ML and BF, by six serial backcrosses (Figure 1), we confirmed the stability of the male bias phenotype and tested additionally five life history parameters, egg number, hatching rate, larval mortality, pupal mortality and adult survival in small cages, during introgression (F1 and BC1 generation) and in the two final introgressed TG_BC6 strains (Table S1) for all crosses reported in Table S2–3. Mean ± Standard Error for each life history parameter *per* cross was reported in Table S2–3 and visualized in Figure S1–S5. All post‐hoc non‐parametric multiple Dunn test results were reported in Figure S1–S5 after providing significance within the generalized linear model (GLM) (Table 1).

**TABLE 1:** Overall significant differences in sex ratio, egg number, hatching rate, larval mortality, pupal mortality (total, male and female) and adult survival in small cages (total, male and female) recorded in the donor transgenic strain Ag(PMB)1, G3 strain from which the transgenic Ag(PMB)1 line was derived, transgenic founder generations (F1), first transgenic backcrossed (BC1) and BC6 transgenic offspring obtained by six serial backcrossing with two recipients wild‐type *An. Coluzzii* strains, Mali‐NIH (ML) and BF_Ac(WT) (BF)

Life history traits	GLM analysis^a^								
		ML genetic background				BF genetic background			
	Distribution of error	F	*χ* ^2^	*df*	*p*‐value	F	*χ* ^2^	*df*	*p*‐value
Sex ratio (TG ♀)	Binomial		7.84	5	0.165 ^ns^		9.29	5	0.098 ^ns^
Sex ratio (TG ♂)	Quasi‐binomial	43.93		2	< 0.001***	76.57		2	< 0.001***
Egg number	Negative‐binomial		96.9	10	< 0.001***		22.1	10	0.0147 *
Hatching rate	Quasi‐binomial	15.29		10	< 0.001***	4.489		10	< 0.001***
Larval mortality	Quasi‐binomial	17.53		10	< 0.001***	5.802		10	< 0.001***
Total pupal mortality	Quasi‐binomial	6.099		10	< 0.001***	2.121		10	0.0308 *
Male pupal mortality	Quasi‐binomial	6.087		10	< 0.001***	7.119		10	< 0.001***
Female pupal mortality	Quasi‐binomial	4.392		10	< 0.001***	2.765		10	0.0052**

	Non‐Parametric Kruskal Wallis test^b^								
		ML genetic background				BF genetic background			
	Distribution of error		*χ* ^2^	*df*	*p*‐value		*χ* ^2^	*df*	*p*‐value
Total adult survival	Not‐Gaussian		2.62	4	0.615 ^ns^		9.14	4	0.051 ^ns^
Male adult survival	Not‐Gaussian		2.92	4	0.564 ^ns^		6.73	4	0.146 ^ns^
Female adult survival	Not‐Gaussian		3.87	4	0.409 ^ns^		6.33	4	0.159 ^ns^

Abbreviation: ns, not significant.

^a^ Generalized linear model (GLM) analysis for five life history traits. Analysis of deviance for multivariate GLM fits using *Chi‐square* test with negative‐binomial and binomial distribution or F test with quasibinomial distribution.

^b^ Significant differences in 50% of adult survival in a small cage between crosses were evaluated using the Non‐Parametric Kruskal Wallis test.

* *p* < 0.05;

** *p* < 0.01;

*** *p* < 0.001.

No significant deviation from the expected transgene‐mediated sex ratio was detected during (F1 and BC1 generation) and after introgression (BC6) using TG females (Table 1, Figure S1). Because TG females do not express the I‐PpoI enzyme, the sex ratio remained unaffected at approximately 50% when TG females mated to non‐TG males. When crossed with non‐transgenic comparators (ML, BF, nonTG_ML_BC6 or nonTG_BF_BC6 females, see Table S2), introgressed TG males (TG_ML_BC6 or TG_BF_BC6) showed a male bias ratio ranging from 98.30 ± 0.3% to 99.0 ± 0.2%. These values were significantly higher than those observed (88.90 ± 0.13%) or previously reported (95.5%) (Galizi et al., 2014) using Ag(PMB)1 males (Table 1, Figure S1). According to two‐way Non‐Parametric Kruskal Wallis tests, we observed no change in egg number, hatching rate, pupal mortality and adult survival between the donor Ag(PMB)1, the introgressed progenies TG_ML_BC6 and TG_BF_BC6 or their non‐transgenic comparator siblings (Table 1, Figure S2–S5). However, the number of eggs, hatching rate and larval survival for the recipient wild‐type ML was lower compared to the introgressed transgenic and non‐transgenic ML_BC6 progeny, Ag(PMB)1 and the non‐transgenic controls G3, indicating this difference is unrelated to the transgene. A large variation in larval mortality was also detected in the introgressed TG_BF_BC6 progeny using the recipient BF strain (Table 1, Figure S3). The larval mortality was significantly higher in crosses involving transgenic and non‐transgenic BF_BC6 mosquitoes in comparison to their parental strains.

### Genotyping the TG_BC6 offspring by DIS assay and AIMs markers

Parental strains and progeny from experimental crosses, for a total of 1400 individuals, were individually genotyped using 15 divergence island SNPs occurring in three regions of genomic divergence proximal to the centromeric region of chromosome X, 2L and 3L. In agreement with Lee et al. (2013), each sample population showed significant linkage disequilibrium between polymorphic SNP loci within each divergence island on the X, 2L and 3L chromosomes (*p* < 0.0001).

Since the 15 selected divergence island SNPs are species‐specific for *An. gambiae* and *An. coluzzii*, the DIS assay allowed us to genotype and distinguish the donor transgenic strain from the wild‐type recipient colonies. Ag(PMB)1 and G3 showed 100% *An. coluzzii*‐type individuals in the 2L island and *An. gambiae*‐X‐linked markers, in both homozygous (Ag(PMB)1 = 66%, G3 = 82%) and heterozygous (Ag(PMB)1 = 28%, G3 = 17%) states. They also included heterozygous admixed individuals (Ag(PMB)1 = 34%, G3 = 39%) and *An. gambiae* homozygous individuals (Ag(PMB)1 = 3%, G3 = 6%) for 3L‐linked SNP markers (Figure 1). Genotypic distribution analysis confirmed that the recipient ML strain was composed of *An. coluzzii* specimens for all 15 SNPs investigated. In contrast, patterns of hybridization between *An. gambiae* and *An. coluzzii* were observed for the recipient wild‐type strain BF in the 2L‐island only. Five *An. gambiae*‐2L‐linked SNPs were detected in 78% of the recipient wild‐type BF specimens (heterozygotes = 55%, homozygotes = 23%) showing typical 3L‐ and X‐island‐*An. coluzzii* markers (Figure 1).

**FIGURE 2:**
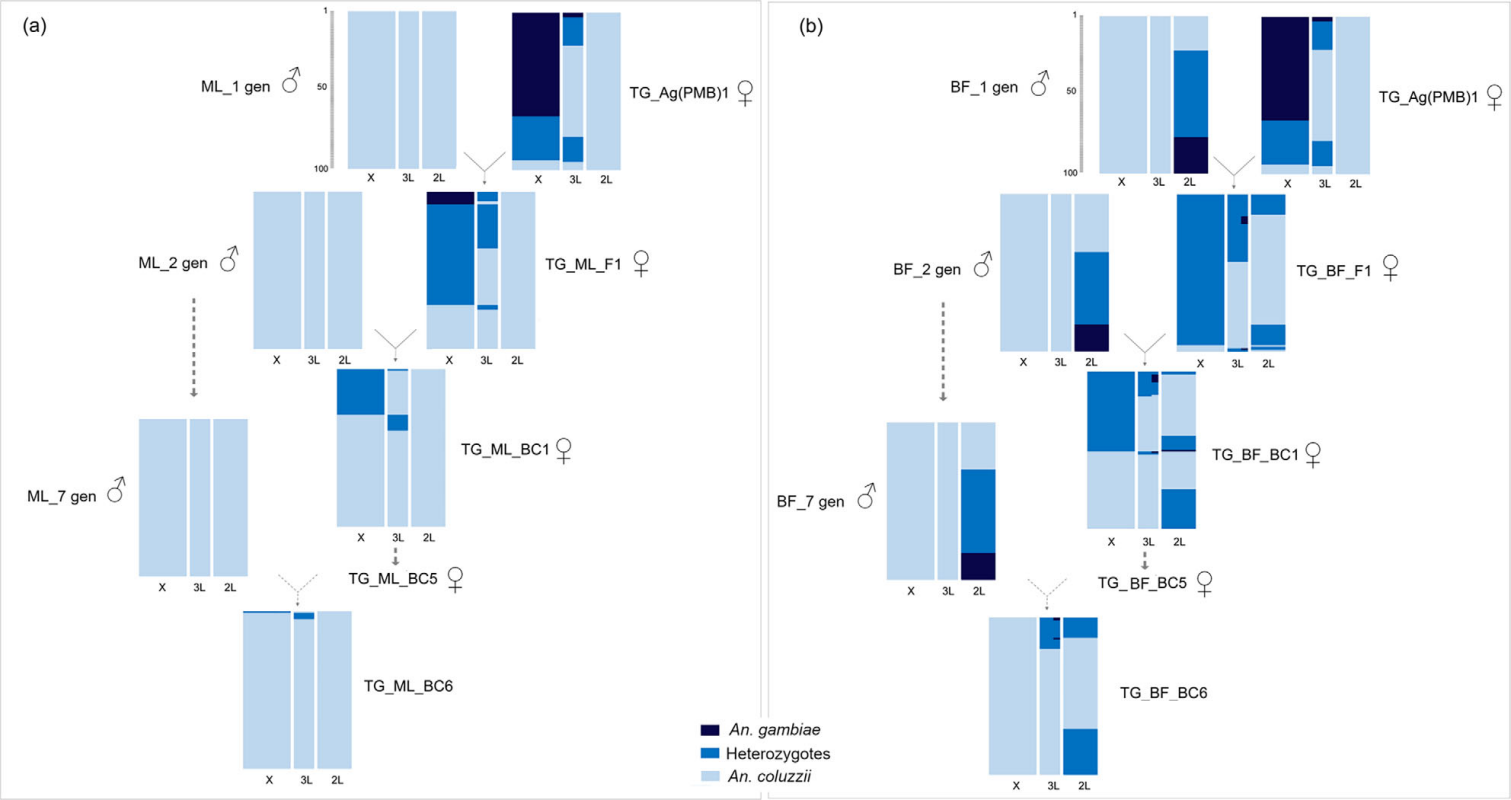
Following the serial introgression of the sex ratio distortion transgene (TG) from Ag(PMB)1 into two *An. Coluzzzii* genetic backgrounds, Mali‐NIH (ML) and BF_Ac(WT) (BF) by DIS assay. Genotypic characterization of the donor parental strain Ag(PMB)1, two wild‐type *An.Coluzzii* recipient colonies, ML and BF at generation 1, 2, and 7, transgenic F1 and BC1 generations produced during introgression processes, the backcrossed transgenic BC6 progenies, using the divergence island SNPs. (DIS assay, Lee et al., 2013). Columns represent 15 discriminative SNPs for *An. Coluzzi* and *An. Gambiae*. For each population, 100 individual mosquitoes are represented by coloured horizontal lines, with individuals stacked vertically.

We evaluated the degree of genomic introgression of the recipient genetic backgrounds into the backcrossed transgenic strains after a series of six backcrosses (Figure 2). Assuming Hardy–Weinberg equilibrium, no significant differences between the expected and observed genotypic frequencies for X‐ (*χ* ^*2*^ = 0.117, *df* = 2, *p* = 0.943), 3 L‐ (*χ* ^*2*^ = 0.541, *df* = 2, *p* = 0.126) and 2L‐linked SNPs were detected in the TG_ML_BC6 progeny (Figure 2a). The DIS analysis of the TG_BF_BC6 progeny revealed a full recovery of the recipient parent genome BF for seven X‐linked SNPs (*χ* ^*2*^ = 3.753, *df* = 2, *p* = 0.153) but statistically significant deviations from the expected genotypic frequencies for the 2L‐SNPs (*χ* ^*2*^ = 38.182, *df* = 2, *p* < 0.0001) and 3L‐SNPs (loci 3L10313‐052 and 3L10315‐679, *χ* ^*2*^ = 371.002, *df* = 2, *p* < 0.0001; locus 3L10317‐546, *χ* ^*2*^ = 265.665, *df* = 2, *p* < 0.0001). An excess of heterozygotes for 3L‐SNPs (20%–17% observed frequencies vs. 1% expected frequencies) and lack of homozygotes for 2L‐*An. gambiae* SNP variants (0% observed frequencies vs. 17% expected frequencies) were observed in the TG_BF_BC6 offspring (Figure 2b).

Finally, a supplemental set of 329 Ancestry Informative Markers (AIMs) with allele frequency differences >0.90 between Malian *An. coluzzii* and *An. gambiae* specimens (Vicente et al., 2017), spread across all chromosome arms, were also screened in our sample dataset using the Pool‐Seq computation (Sup. Information ‘Whole genome pooled sequencing’). The frequency analysis of AIMs reinforced the DIS Assay outputs for the backcrossed transgenic BC6 progenies and confirmed the genetic admixture between *An. gambiae* and *An. coluzzii* for the donor transgenic strain Ag(PMB)1 and G3, with a ~ 4.2 Mb stretch on the X chromosome (X‐chromosome_15100037:19305628) of ‘pure’ *An. gambiae* AIM variants adjacent to the pericentromeric region (Figure S6–S7). As expected, despite an overall genomic prevalence of *An. coluzzii*‐type‐AIMs, the recipient wild‐type strain BF showed a high number of 2L‐linked AIMs, 31.5% at generation 1 and 24% at generations 2 and 7, with *An. gambiae*‐type‐frequency ranging from 0.45 to 0.75 (Figure S6–S7).

### Pattern of genome‐wide differentiation in the TG_BC6 offspring

The degree of genome‐wide introgression of the ML and BF recipient genetic background into the two backcrossed transgenic BC6 progenies was also evaluated by using the Whole Pool‐Seq genome scans (Sup. Information ‘Whole genome pooled sequencing’).

**FIGURE 3:**
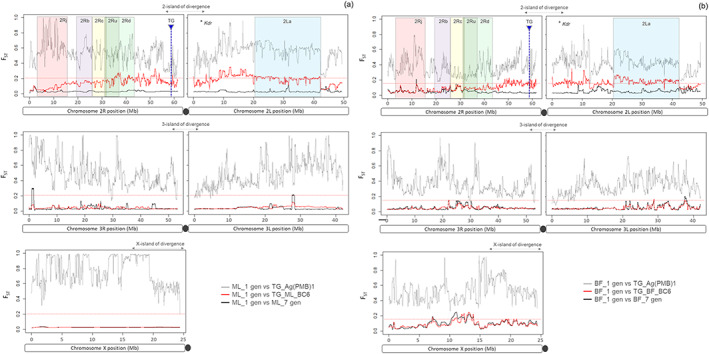
The genomewide F_ST_ differences on the chromosomes 2, 3 and X between parental and introgressed transgenic strains in two genetic backgrounds (ML and BF). Smoothed F_ST_‐values for (a) the wildtype recipient strain ML at generation 1 (ML_1 gen) and (b) the wildtype recipient strain BF at generation 1 (BF_1 gen) versus the transgenic donor strain Ag(PMB)1, the backcrossed transgenic BC6 progenies and the recipient wildtype strains at generation 7 used as an internal control. The 1 kb sliding window‐based approach was used to generate the F_ST_‐values for each chromosome arm. The 85% threshold for each F_ST_ distribution is indicated with a dashed red line. Six most common inversions of chromosome 2 (2Rj, 2Rb, 2Rc, Ru, 2Rd and 2La) are shown by the coloured‐shaded areas. The three islands of divergence, the transgene integration site (TG) and *Kdr* locus were also reported at the top of the figure.

The kernel density plots of the genetic differentiation F_ST_ distributions calculated over 1 kb sliding windows for each chromosome arm highlighted a differential introgression rate for chromosome 2 compared to chromosomes 3 and X in both transgenic introgressed BC6 strains (Figure S8 and Table S4). Inter‐parental‐strain genomic differentiation was generally high for autosomes (mean F_ST_ ≥0.36 ± 0.158), with a maximum divergence for X‐chromosome (Ag(PMB)1 versus ML_1 gen = 0.796 ± 0.158, Ag(PMB)1 vs. BF_1 gen = 0.536 ± 0.197). After introgression, all TG_BC6 versus recipient wild‐type strain F_ST_ distributions were highly positively skewed and leptokurtic and low mean genomic differentiation was observed in three chromosome arms, X (TG_ML_BC6 = 0.030 ± 0.029; TG_BF_BC6 = 0.099 ± 0.070), 3L (ML_TG_BC6 = 0.051 ± 0.028; BF_TG_BC6 = 0.049 ± 0.027) and 3R (TG_ML_BC6 = 0.042 ± 0.025; TG_BF_BC6 = 0.053 ± 0.034), in comparison to the respective recipient wild‐type strain (Figure S8 and Table S4). The average genomic divergence of the 2R chromosome arm slowly declined after six serial backcrosses with the recipient strains ML (TG_ML_BC1 = 0.166 ± 0.068; TG_ML_BC6 = 0.144 ± 0.075) and BF (TG_BF_BC1 = 0.105 ± 0.059; TG_BF_BC6 = 0.091 ± 0.063). The 2L chromosome arm exhibited much greater genomic divergence in both backcrossing processes, showing a near‐identical level of mean F_ST_ differentiation in TG_BC1 (TG_ML_BC1 = 0.216 ± 0.080; TG_BF_BC1 = 0.173 ± 0.075) and TG_BC6 offspring (TG_ML_BC6 = 0.192 ± 0.082; TG_BF_BC6 = 0.166 ± 0.071). Manhattan plots of the genomewide F_ST_ values computed between the wildtype recipient strains versus the backcrossed transgenic BC6 offspring displayed numerous small windows of significant genomic divergence at irregular intervals along all chromosomes (Figure 3). Differences in SNP frequency were especially pronounced in a large region of chromosome 2, ~89 Mb and ~ 70 Mb long for TG_ML_BC6 and TG_BF_BC6 respectively, which included the *2La* polymorphic chromosome inversion extending from approximately 20–42 Mb genomic position on the 2L arm, the centromere and then ~7.7 Mb‐2Rb, ~4.7 Mb‐2Rc, ~4.0 Mb‐2Ru and ~ 10.8 Mb‐2Rd polymorphic inversions on the 2R arm, respectively (Figure 3).

Clustering tree analysis confirmed the genetic similarity of TG_ML_BC6 and TG_BF_BC6 with the respective wildtype recipient colony based on genomewide polymorphic SNPs of chromosome X and both arms of chromosome 3 (Figure S9–S10). Using only the subsets of SNPs for chromosome arm 2L and 2R, the serial transgenic offspring F1, BC1 and BC6 of both ML and BF strains, formed a discrete genetic group, showing low recovery of the recipient parent genome in chromosome 2 after six backcrosses.

### Introgression of 2L and 2R chromosomal inversions in TG_BC6 offspring

**FIGURE 4:**
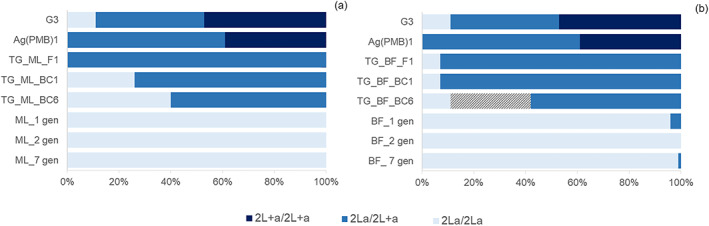
Distribution of the 2La chromosome inversion genotypes (homozygotes 2L+^a^/2L+^a^, heterozygotes 2La/2L+^a^ and homozygotes 2La/2La) assayed by PCR (White et al., 2007) during the serial backcrossing of transgene from Ag(PMB)1 into two *An. Coluzzii* genetic backgrounds (ML and BF). Karyotype frequencies (%) of the 2La chromosome inversion in the two wildtype recipient strains ML (a) and BF (b) at generation 1, 2 and 7, the transgenic donor strain Ag(PMB)1, G3 control, transgenic F1 and BC1 generations produced during introgression processes and the backcrossed transgenic BC6 progenies. The increment of 2La/2La frequency in the transgenic mosquitoes introgressed with Burkina Faso genetic background at generation number 22 (TG_BF_BC22) is also displayed in the striped area.

The Pool‐Seq‐screening of five tag SNPs identified as predictive markers of inversion orientation for five common polymorphisms in chromosome 2 (2Rb, 2Rc, 2Ru, 2Rd and 2La) (Love et al., 2019), revealed that polymorphism of the 2La inversion was mainly the cause of the large genomic differentiation observed on the 2L arm in both ML and BF TG_BC6 offspring, as well as 2Rb, 2Rc, 2Ru and 2Rd inversions in TG_ML_BC6 offspring only (Figure S11). In contrast to BF strain showing a similar frequency distribution of inversion polymorphisms in comparison to the donor Ag(PMB)1 for the chromosome arm 2R, the recipient wildtype ML resulted to be monomorphic for the 2Rb, 2Rc, 2R+^u^ and 2R+^d^ chromosomal variants at any generation. The donor 2Rb, 2Rc, 2R+^u^, 2R+^d^ arrangements progressively increased in frequency in TG_ML_BC6 but not to frequencies as it would be expected for Hardy–Weinberg equilibrium after six backcrosses (*χ* ^2^ ≥ 46.33, *df* = 1, *p* ˂ 0.0001). No significant departures from the Hardy–Weinberg expectations were detected for tag SNPs correlated with 2Rb (*χ* ^2^ = 0.874, *df* = 1, *p* = 0.353), 2Rc (the donor and BC6 are identical), 2Ru (χ^2^ = 0.219, *df* = 1, P = 0.638) and 2Rd (*χ* ^2^ = 0.838, *df* = 1, *p* = 0.369) inverted regions in the TG_BF_BC6 progeny (Figure S11). Along with the exploitation of tag SNPs, molecular genotyping of 1400 individual mosquitoes by PCR diagnostic assay (White et al., 2007) confirmed that prior to the backcrossing process, the 2La arrangement was segregating at a frequency of 30.5% (39% 2L+a/+a; 61% 2La/+a) and 98% (4% 2La/+a, 96% 2La/a) in the donor transgenic Ag(PMB)1 and the recipient wildtype BF at the first generation. The recipient wildtype ML also resulted to be fixed for the 2La chromosomal variant (Figure 4). After six serial backcrosses, the 2La arrangement slowly increased in frequency up to 70% (60% 2La/+a; 40% 2La/a) and 55.5% (89% 2La/+a; 11% 2La/a) in TG_ML_BC6 and TG_BF_BC6 respectively, without reaching almost fixation (1% 2La/+a; 99% 2La/a; *χ* ^2^ ≥ 231, *df* = 2, *p* ˂ 0.0001). After 22 backcrosses to recipient BF, the 2La inverted arrangement still persisted at frequency of 71% (58% 2La/+a; 42% 2La/a) in the final TG_BF_BC22 progeny (Figure 4).

### *Kdr* mutation frequencies in TG_BC6 offspring

The frequencies of the L1014F [TTA → TTT] and L1014S [TTA → TCA] point mutations, which confer knockdown resistance (kdr) to DDT and pyrethroids (Santolamazza et al., 2008) were also inferred in our datasets. Whilst the L1014F *kdr_r* variant was not found in the donor Ag(PMB)1 and recipient wildtype ML and therefore in their subsequent ML‐backcrossed offspring, the *kdr_r* was mapped at 38.2%–48.8% in the recipient wildtype BF at each generation (Figure S12). Therefore, the *kdr_r* resistant allele segregated in the transgenic BF_F1 (12.5%) and BF_BC1 generations (17.2%) and the backcrossed transgenic BF_BC6 offspring (25.0%), and was positively correlated to the *An. gambiae* species‐specific haplotype in the 2L divergence island (*r* ^2^ = 0.925) (Figure S12). In this BF genetic background, the frequencies of five SNP markers tagging the haplotype‐A bearing the *kdr_r* allele and associated with susceptibility to *P. falciparum* within the 2L island were inferred in the transgenic offspring (Mitri et al., 2015). Similar to the L1014F *kdr_r* variant, Haplotype A (HapA)‐SNP frequencies only found in BF genetic background gradually raised in the transgenic offspring (BF_F1 = 0.144 ± 0.011; BF_BC1 = 0.175 ± 0.027; BF_BC6 = 0.233 ± 0.025), without reaching the relatively high frequencies of the recipient BF (generation 1 = 0.594 ± 0.018; generation 2 = 0.394 ± 0.029; generation 7 = 0.419 ± 0.030) (Figure S12). L1014S polymorphism was not detected across our sample collections.

## DISCUSSION

In this study, the introgression of a synthetic sex‐ratio distortion transgene from the donor Ag(PMB)1 strain by six serial backcrosses into two recipient colonies of *An. coluzzii* demonstrated that the male bias has been maintained in distinct genetic backgrounds. Our results also highlight the varying introgression rates of the wild‐type genomes across the three chromosomes. The chromosomes X and 3 from ML and BF wild‐type genetic backgrounds were completely introgressed, whereas chromosome 2 and specifically the chromosome arm 2L kept a large (~85%) genomic footprint from the donor strain, even after six backcrosses, probably partly due to the genomic location of the transgene. Assuming Hardy–Weinberg equilibrium six backcrosses should ensure around 99.22% recovery of the recipient parent genome in each of the new introgressed TG strains.

### The donor and recipient strains

Irrespective of genotyping analysis, we confirmed that donor Ag(PMB)1, which was generated by transforming the G3 colony, was isolated in 1975 in The Gambia and initially classified as *An. gambiae s.s*. is composed of hybrid specimens with mixed features from both *An. gambiae* and *An. coluzzii*, but with *An. gambiae* species‐specific markers mainly on chromosome X. This is fully consistent with the identification of asymmetrical hybrid forms characterized by an admixed autosomal genome and *An. gambiae* like X‐pericentromeric islands in The Gambia and Guinea Bissau, where stable elevated hybrid rates have been reported (Pombi et al., 2017). The peculiar features of West Africa (e.g., elevated breeding site salinity//brackishness and competition with euryhaline *Anophele melas* sibling species) might have favoured the evolution of new hybrids with common centromeric genomic regions at the X chromosome of *An. gambiae*, harbouring factors responsible for reproductive isolation (Caputo et al., 2016; Vicente et al., 2017).

The genotypic scan of DIS‐Assay SNPs and AIMs across all chromosome arms also proved that the recipient wild‐type strain ML, sampled at Niono (Mali) in June 2005, is composed by *An. coluzzii* specimens while, conversely, BF mosquitoes collected in Vallée Du Kou (Bama, Burkina Faso) in 2014, are admixed for the 2L‐island linked markers. Genotypic profiles similar to BF individuals were attested in *An. coluzzii* populations of Ghana (Clarkson et al., 2014), Mali (Norris et al., 2015) and Burkina Faso (Hanemaaijer et al., 2019). After bursts of hybridization in 2006, *An. coluzzii* inherited from *An. gambiae* the entire 2L genomic island, which includes the knockdown resistance gene variants *kdr*. This natural adaptive introgression was coincident with the start of major insecticide‐treated bed net (ITN) campaigns in Western Africa. The observed admixed mosquitoes for the 2L genomic island and the detection of L1014F *kdr* mutation at moderately high frequency in the recipient BF colony can be ascribed to the progressive adaptive introgression of the L1014F *kdr* mutation from *An. gambiae* to *An. coluzzii* as a result of increased insecticide exposure, which acted as a selective force in *Anopheles* mosquito populations in Burkina Faso (Hanemaaijer et al., 2019).

### Successful introgression of the sex distorter transgene in two recipient *An. Coluzzii* genetic backgrounds

The introgression of the non‐gene drive sex distorter [3xP3‐DsRed]β2‐eGFP::I‐PpoI‐124L from the donor Ag(PMB)1 into two recipient *An. coluzzii* genetic backgrounds, ML and BF, by six consecutive backcrosses demonstrated the capability of I‐PpoI to recognize and cleave its 15 bp target sequence present within the 28 S rDNA unit clustered in 500–700 copies (Gentile et al., 2002) on the fully‐introgressed *An. coluzzii* X chromosome, during male meiosis. Previously, the same [3xP3‐DsRed]β2‐eGFP::I‐PpoI‐124L transgene was introgressed into the sibling vector species *Anopheles arabiensis*, in which the construct was shown to induce paternal male bias in the recipient strain (Bernardini et al., 2019). Similarly, male bias in the progeny of TG_ML_BC6 and TG_BF_BC6 introgressed males was high. In addition, life history parameters of I‐PpoI introgressed mosquitoes were not significantly different from the donor and wild‐type recipient strains, except for the higher larval mortality of BF_BC6 transgenic/not‐transgenic males than the donor BF and the recipient ML showing lower female/male fertility and larval survival than transgenic/not‐transgenic ML_BC6 offspring. The latter results suggested that the ML colony could start suffering from inbreeding depression due to low effective population sizes, with some fitness costs in comparison to BF_BC6, considering that the ML strain has been maintained and reared for ~16 years in laboratory conditions. The difference in fitness observed in larval mortality between the introgressed BF_BC6 males and BF recipient is not related to the transgene due to the absence of statistical significance in larval mortality between TG_BF_BC6 and nonTG_BF_BC6 sibling. Therefore, it could be dependent either on laboratory conditions or on the genomic background of BC6 generation which may differ after continuous backcrossing to wild‐type. Larval mortality differences are worth to be further explored for addressing any potential implications for the aquatic stage of the transgenic mosquitoes to survive in the field.

### Differential genomic introgression rates in the final transgenic backcrossed strains

In the absence of natural selective pressure, patterns of differential introgression have been detected across the entire genome of the BC6 transgenic offspring. Although genetic drift resulting from the small population size of lab‐adapted strains could have augmented the genetic differences already existing between the donor Ag(PMB)1 and the two recipient wild‐type *An. coluzzii* colonies isolated in Mali and Burkina Faso in 2005–2014, respectively, a similar general pattern of widespread genomic divergence has been observed in the two introgressed transgenic strains after six serial backcrosses using indoor‐small cage settings. Despite the extensive autosomal adaptive introgression, less pervasive on sex‐chromosome X, observed between *An. coluzzii* and *An. gambiae* field populations (Caputo et al., 2016), we highlighted a remarkable genomic divergence in a large region of chromosome 2 (≥ 85% threshold for each F_ST_ distribution) between the BC6 transgenic offspring and the recipient paternal strains. This genomic block includes an approximately 35‐Mb pericentromeric region, characterized by extremely low recombination rates (White et al., 2010). Coupled with the transgene location in the band 2R‐19D, near the centromere of chromosome 2 (Vitale et al., 2022) and the high linkage disequilibrium of the neighbouring pericentromeric environment, the artificial selection of the adult females carrying the transgene as maternal donors at each stage of the serial backcrossing strategy may have contributed to retain G3 haplotypes in such a genomic region. The incomplete introgression of the *kdr_r* resistant allele associated with the BF‐specific 2L‐island haplotype in the final transgenic BF_BC6 offspring is consistent with this prediction.

High genomic differentiation was also detected between the introgressed transgenic strains and the recipient colonies in the ~22 Mb‐large 2La chromosomal inversion in which crossing‐over between alternative arrangements of heterokaryotypes is effectively suppressed (Stump et al., 2007), as well as between TG_ML_BC6 and ML for four paracentric inversions of right arm 2Rb, 2Rc, 2Ru and 2Rd. Indeed, as proved by individual PCR assay genotyping, 2La/2L+^a^ heterozygosity persisted at much higher rates than theoretically expected in the left arm. Similar heterozygote excess in violation of Hardy–Weinberg expectations has been observed in the 2La region of an *An. coluzzii* inbred line (Turissini et al., 2014), sometimes in‐field and laboratory populations within the *An. gambiae* complex (Brooke et al., 2002; Tennessen et al., 2021). In all these cases, associative overdominance has been evoked as a plausible explanation for the retention of such polymorphism. Under this assumption, tight linkage disequilibrium between neutral loci and loci subject to selection, either against partially recessive alleles or in favour of heterozygotes, might have promoted heterozygote advantage. It is nonetheless important to note that 2La inversion is associated with adaptation to levels of aridity along ecoclimatic clines, with 2La approaching fixation in the xeric habitats, and 2L+^a^ predominating in the humid rainforests (Vicente et al., 2017). Although an independent assortment of left and right chromosome arms is assumed, the inversions 2La, 2Rb and 2Rc exhibit common eco‐geographical patterns in *An. gambiae* and *An. coluzzii* (Ayala et al., 2017). Considering the superior resistance to desiccation and thermal stress conferred by 2La arrangement in lab mosquitoes at the adult and larval stages, respectively, with the recently demonstrated epistatic effect of the 2Rbc combination (Ayala et al., 2019; Cheng et al., 2018), we cannot rule out that the laboratory ‘benign‐wet’ conditions might have altered the introgression efficiency of 2La and one or more 2R arrangements in the TG_BC6 offspring.

### Plausible implications

Using TG_BC6 offspring as a paradigm for the evaluation of candidate GMM strains selected for progression to any level of field testing, we postulated that the lack of complete introgression in some genomic regions of chromosome 2 could have multiple implications for disease transmission as well as ecological adaptation. High levels of polymorphism for three paracentric inversions, Rb, 2Rc and 2La, might serve as allelic reservoirs conferring TG_BC6 mosquitoes’ ecological flexibility and reinforcing their adaptive potential for exploiting different habits across sub‐Saharan regions (Ayala et al., 2019; Cheng et al., 2018). However, the persistence of the 2L+^a^ variant might increase the susceptibility of the TG_BC6 mosquitoes to the *P. falciparum* pathogen in comparison to the recipient wild‐type strains. The inverted 2La region has been found in fact to be associated with a lower malaria oocyst infection prevalence and intensity (Riehle et al., 2017). In addition, mosquitoes carrying the more‐susceptible allele (2L+^a^) might also show a reduced tendency to rest indoors and feed inside habitations, avoiding the indoor‐based vector control tools such as ITNs and indoor residual spraying (IRS) (Riehle et al., 2017). Conversely, the incomplete introgression of the *An. gambiae*‐specific 2L‐island haplotype in the TG_BF_BC6 progeny might mitigate the epidemiological effects due to the presence of *kdr_r* resistant alleles in the wild‐type recipient BF. It is also worth noticing that a decreased immune refractoriness to the *P. falciparum* parasite has been demonstrated in *kdr*‐resistant mosquitoes likely caused by an immune gene, serine protease *ClipC9*, associated with the HapA within the 2La‐island, ~291 kb apart from the L1014F point mutation (Mitri et al., 2015). As a regulatory step for the evaluation of the potential risk of GMM mosquitoes, the ‘net effect’ outcomes of the identified genomic divergences between the TG_BC6 and the recipient wild‐type strains in terms of vector competence, fitness and insecticide resistance, coupled with population suppression effects, should be assessed thoroughly. Given the complex scenario of the net effect putative outcomes, the extension of the backcrossing breeding strategy beyond BC6 generation should be a pre‐requirement for any candidate strain for field release. Notably, the 2L^+a^ arrangement still persisted at a frequency of 29% after 22 consecutive backcrossing events in the TG_BF_BC22 progeny.

This data suggests the additional need to force the introgression of critical regions that have ecological and epidemiological implications through a selection of specific genetic markers targeting those regions as part of the backcrossing scheme. We propose to design a PCR‐marker‐assisted crossing scheme for obtaining the desired 2‐chromosome configuration already in the ≥ BC1 generation and thereby overcome the introgression barrier at chromosome 2 in future studies. The proposed PCR‐marker‐assisted crossing scheme together with the demonstrated functionality of the transgene will support the application of genetic vector control strategies with self‐limiting GM mosquitoes into *An. coluzzii* populations in West Africa. We recommend that the integration of the whole‐genome sequencing data with phenotypic information (e.g., fitness costs, vector competence and insecticide resistance) of the introgressed transgenic strain should be a key requisite for designing genetic vector control strategies. This will prevent the release of novel combinations of genetic material in comparison to the local wild‐type counterparts whose potential action could not be accurately predicted into the local mosquito populations or any change that increases the mosquito's ability to transmit malaria.

## EXPERIMENTAL PROCEDURES

### Mosquito strains and transgene introgression

The Ag(PMB)1 transgenic line was developed from the G3 wildtype colony and is classified as a sex ratio distorter ‐ autosomal X‐shredder based on I‐PpoI homing endonuclease (Galizi et al., 2014). Due to the sex ratio distortion conferred by the [3xP3‐DsRed]β2‐eGFP::I‐PpoI‐124L transgene, Ag(PMB)1 strain has been maintained in the laboratory by continuously backcrossing Ag(PMB)1 females to G3 males. For this reason, we expect the two lines to differ only in terms of the presence or absence of the transgene. The transgenic mosquitoes were identified by screening for the expression of the 3XP3‐DsRed transgene marker visible in the thoracic, abdominal ganglia and optic lobes.

From 2018 to 2019, we introgressed the [3xP3‐DsRed]β2‐eGFP::I‐PpoI‐124L transgene from the donor parent Ag(PMB)1 into the genetic background of two recipient *An. coluzzii* wild‐type colonies: (experiment 1) Mali‐NIH (ML) sampled at Niono (Mali) in June 2005 (stock number MRA‐860) and (experiment 2) BF_Ac(WT) (BF) collected in Vallée Du Kou (Bama, Burkina Faso) in 2014 by the Institut de Recherche en Sciences de la Santé (IRSS, Bobo‐Dioulasso) (Table S1). The latter specimens were preliminarily identified as *An. coluzzii* individuals by PCR‐based diagnostics targeting species‐specific polymorphisms at the intergenic spacer (IGS, 540th and 649th nucleotide position) of the 28 S ribosomal DNA (Fanello et al., 2002). All mosquito strains were reared and maintained at standard mosquito colony conditions of 28 ± 1°C and 80% ± 10% relative humidity with the light and dark regime as described in Facchinelli et al. (2019): 45 min sunset, 11:15 h night, 45 min sunrise and 11:15 h full light. In each introgression experiment, we performed an initial cross between the transgenic donor and recurrent wild‐type parent. In brief, we established two small cages (17.5 × 17.5 × 17.5 cm, MegaView Science Co., Ltd., Taiwan), each containing 150 Ag(PMB)1 transgenic females and 150 *An. coluzzii* wild‐type males (Figure 1). Adult mosquitoes were allowed to mate for seven days before blood‐feeding them with defibrinated and heparinized sterile cow blood (Allevamento Blood di Fiastra Maddalena, Teramo, Italy) using a Hemotek membrane feeder (DiscoveryWorkshops, Accrington, UK). Eggs were collected from both cages, counted (Egg‐Counter v1.0 software) and mixed to provide the F1 founder generation. The resultant transgenic F1 individuals were crossed to the recipient parent to develop a backcross one (BC1) generation. By the same method, three hundred transgenic adult females were randomly selected from F1 hybrid offspring and used for the first backcross (BC1) with *An. coluzzii* wild‐type males in two small cages. Transgenic individuals from the BC1 generation were once again crossed to the same recurrent strain. The introgression of the transgene to recipient wild‐type parent *An. coluzzii* strain was continued by backcrossing the subsequent transgenic female offspring to the paternal *An. coluzzii* wild‐type strain for a further five times to generate the BC6 transgenic (TG) mosquitoes (Figure 1). Each backcrossing generation includes both transgenic (heterozygous for the transgene) and non‐transgenic individuals. The transgenic individuals were discriminated against by screening for the expression of the 3XP3‐DsRed transgene marker for the next generation of back‐crossing.

### Life history parameters

Life history parameters, such as sex ratio, egg number, hatching rate, larval mortality, pupal mortality and adult survival in a small cage, were investigated in the parental strains, G3 control, intermediate F1 and BC1 transgenic generations and in the introgressed transgenic strains at generation BC6 as described in Pollegioni et al. (2020). For testing statistical differences of the first five life history parameters between the different crosses, we applied a generalized linear model (GLM) approach with the binomial, quasi‐binomial and negative‐binomial distribution of errors and a logit link function, as implemented in the R package *glm2* and *MASS*. Following the statistical significance within the GLM, the post‐hoc non‐parametric multiple Dunn tests were performed to investigate two‐way differences between crosses in terms of sex ratio, egg number, hatching rate, larval and pupal mortality using the *R*‐*dunn.test* package. Finally, mosquito survival curves were analysed using Kaplan–Meier Survival analysis, and log‐rank tests were used to determine the equality of the survival distributions between replicates for each cross (*R‐survival* package v2.37–4). Variation in the median survival was analysed by the non‐parametric Kruskal‐Wallis rank sum test. All tests were computed using R v3.4.0 statistical programming language (R Development Core 2017).

### DIS genotyping assay

The DIS genotyping assay consists of 15 single nucleotide polymorphisms occurring in three small regions of genomic divergence proximal to the centromeric region of chromosome X (7 SNPs spanning 4.4 Mbp), 2L (5 SNPs spanning 2.2 Mbp) and 3L (3 SNPs spanning 117 kbp) (Lee et al., 2013). These SNPs were reported to be fixed for *An. gambiae* and *An. coluzzii* species were selected for genotyping the donor transgenic strain Ag(PMB)1, G3 wild‐type control strain, the recipient wild‐type strains ML and BF at generation 1, 2 and 7, the intermediate transgenic F1 and BC1 individuals, and the two backcrossed transgenic BC6 progenies (Figure 1). One hundred adult virgin females were randomly collected from each of 14 mosquito groups, for a total of 1400 individuals (Table S1). Genomic DNA of each individual mosquito was extracted using DNeasy Blood & Tissue Kit (Qiagen, Hilden, Germany), following the manufacturer's instructions. After genomic DNA quantification by Qubit fluorometer (dsDNA, BR, Invitrogen), each individual mosquito was genotyped by the competitive allele‐specific PCR (KASP) platform following the manufacturer's instruction optimized for DIS markers (LGC Genomics Ltd. Hoddesdon, United Kingdom; http://www.lgcgroup.com/products/kasp-genotyping-chemistry). Assuming Hardy–Weinberg equilibrium, we compared the expected and observed genotype frequencies for X, 2L and 3L species‐specific SNPs (homozygotes *An. gambiae*/*An. gambiae*, heterozygotes *An. gambiae*/*An. coluzzii* and homozygotes *An. coluzzii*/*An. coluzzii*) in the introgressed transgenic strains after six backcrosses (TG_ML_BC6 and TG_BF_BC6) using the *Chi‐Squared* Test. We also tested for linkage disequilibrium between DIS markers within each island of divergences using a likelihood ratio test implemented in ARLEQUIN v.3.11 (Excoffier et al., 2007), with 1000 permutation, five initial EM conditions and unphased genotypic data.

### Whole genome pooled sequencing

In order to evaluate the degree of genomewide introgression of the recipient wild‐type strains in the two backcrossed transgenic BC6 progenies, we performed whole‐genome pooled sequencing on the same set of mosquito samples following the Pool‐Seq approach (Futschik & Schlötterer, 2010).

After genomic DNA quantification by Qubit fluorometer (dsDNA, BR, Invitrogen), equal amounts of DNA from 100 individuals per group were pooled, resulting in a pool size of 200 ng of RNA‐free genomic DNA per population. Eight (Ag(PMB)1, G3, ML at generation 1, 2 and 7, TG_ML_F1, TG_ML_BC1 and TG_ML_BC6) and six (BF at generation 1, 2 and 7, TG_BF_F1, TG_BF_BC1 and TG_BF_BC6) libraries were separately prepared using the TrueSeq DNA nano kit and TruSeq dual indexing barcodes (Illumina) and sequenced to an expected mean coverage of 30X using Illumina NextSeq550 platform with paired‐end, 150‐bp reads. DNA libraries were prepared and sequenced at Polo d'Innovazione di Genomica, Genetica e Biologia Società Consortile R.L., Siena, Italy. The genetic differentiation index (F_ST_) was then calculated using the scripts fst‐sliding.pl in POPOOLATION2 software (Kofler et al., 2011) using a sliding‐window approach with a window size of 1000 bp and step size 1000 bp. The bioinformatics data processing with POPOOLATION2 software and the construct of genetic distance trees by using PHYLIP v.3.68 (Felsenstein, 2005) are fully described in detail in Sup. Information ‘Whole genome pooled sequencing’.

In addition, a supplemental set of 329 informative Ancestry Informative Markers (AIMs) located in the chromosome X (236 SNPs), 2R (21 SNPs), 2L (38 SNPs), 3R (24 SNPs) and 3L (10 SNPs), with allele frequency differences >0.90 between Malian *An. coluzzii* and *An. gambiae* specimens (Vicente et al., 2017), were screened in our pool datasets. We build up the mpileup and the synchronized files, inclusive of all the eight pools per introgression experimentS 1 and 2 (Table S1). The allele frequency distribution for each AIM was estimated across pools with function snp‐frequency‐diff.pl implemented in POPOOLATION2 (rc files).

### Frequencies of the 2R and 2La chromosome inversions

The frequencies of tag SNPs for 2Rb, 2Rc, 2Ru, 2Rd and 2La inversion in *An. gambiae* and *An. coluzzii* (Love et al., 2019), were inferred from the allele frequency rc file of chromosome 2 for each introgression experiment. Five robust tags showing high degree of concordance between SNPs and inversion genotype, present in our dataset were selected: tag SNP_2Rb_20,247,651_G/A (Ref_2R+^b^ = G, Alt_2Rb = A, concordance >0.95), tag SNP_2Rc_col_27,282,330_C/T (Ref_2R+^c^ = C, Alt_2Rc = T, concordance >0.80), tag SNP_Ru_31,516,240_C/T (Ref_2R+^u^ = C, Alt_2Ru = T, concordance >0.70), tag SNP_Rd_36,196,095_C/T (Ref_2R+^d^ = C, Alt_2Rd = T, concordance >0.75) and tag SNP_2La_41,966,114_C/T (Ref_2L+^a^ = C, Alt_2La = T, concordance>0.995) (Love et al., 2019). In addition, the 2La inversion karyotype for 1400 individual mosquitoes from two introgression experiments was performed using a molecular PCR diagnostic that spans the inversion breakpoints (White et al., 2007).

### Frequency of the *kdr* and *HapA* mutations

The frequencies of the L1014F [TTA → TTT] and L1014S [TTA → TCA] point mutations, which confer knockdown resistance (kdr) to DDT and pyrethroids (Santolamazza et al., 2008), were extrapolated from the allele frequency rc files of 2L chromosome arm for each introgression experiment. The L1014S and L1014F mutations map 2,422,651 bp and 2,422,652 bp from the centromere respectively, within the genomic island of divergence on chromosome arm 2L, in the AGAP004707 para voltage‐gated sodium channel gene (genome position AgamP4_2L:2,358,158‐2,431,617). In the same region, frequencies of five diagnostic SNPs, tagging the Haplotype‐A bearing the kdr_r allele and associated to susceptibility to P. falciparum (Mitri et al., 2015), were also computed at the following genomic positions: 2L_1272741 [T/C], 2L_1834476 [A/G], 2L_2081228 [T/C], 2L_2430786 [C/T] and 2L_2489212 [T/C].

## CONFLICT OF INTEREST

The authors have no conflict of interest to declare.

## Supporting information


**Appendix S1.** Supporting information.

## Data Availability

Experimental raw data and DNA Pool‐Seq sequence files were submitted to Mendeley Data Repository, Reserved DOI: 10.17632/b7f3c9s5n6.1 and The European Nucleotide Archive (ENA), Project Number PRJEB48699, accession number of fastq files (ERR7457686‐ERR7457699).
