# Supplementary material for: Introgression of a synthetic sex ratio distortion transgene into different genetic backgrounds of Anopheles coluzzii
Source: Insect Mol Biol. 2022 Oct 31;32(1):56–68. doi: 10.1111/imb.12813 (PMC10092091; doi:10.1111/imb.12813)
Supplement: Supplementary file 1 — Appendix S1. Supporting information. [file IMB-32-56-s001.pdf]

Supporting Information

Table S1. List of acronyms and number of samples (N°) used in this study for the donor transgenic strain Ag(PMB)1 (transgenic construct in G3 strain), transgenic generations F1 (founder generation), BC1 (first transgenic backcrossed offspring), BC6 (transgenic offspring obtained after six serial backcrossing) and two recipient wild-type *An. coluzzii* strains, Mali-NIH and BF_Ac(WT).

	Acronyms	Description	N°
**Parental strains**			
Ag(PMB)1	Ag(PMB)1	Donor transgenic line in G3 genetic background.	100
G3	G3	*An.gambiae* s.l wildtype lab colony isolated in 1975 in The Gambia	100
Mali-NIH	ML	Recipient wild-type *An. coluzzii* colony sampled at Niono (Mali) in June 2005.	
	ML_1 gen	ML generation used for producing F1 founder offspring	100
	ML_2 gen	ML generation used for producing first BC1 backcrossed offspring	100
	ML_7 gen	ML generation used for producing BC6 backcrossed offspring	100
BF_Ac(WT)	BF	Recipient wild-type *A. coluzzii* colony sampled in Vallée Du Kou (Burkina Faso) in 2014.	
	BF_1 gen	BF generation used for producing F1 founder offspring	100
	BF_2 gen	BF generation used for producing first BC1 backcrossed offspring	100
	BF_7 gen	BF generation used for producing BC6 backcrossed offspring	100
**Experimental crosses**			
F1	TG_F1	Transgenic F1 founder generation	
	TG_ML_F1	TG_F1 obtained by crossing Ag(PMB)1 transgenic females with ML_1 gen wild-type males	100
	TG_BF_F1	TG_F1 obtained by crossing Ag(PMB)1 transgenic females with BF_1 gen wild-type males	100
BC1	TG_BC1	First transgenic backcrossed BC1 generation	
	TG_ML_BC1	TG_BC1 obtained by crossing TG_ML_F1 transgenic females with ML_2 gen wild-type males	100
	TG_BF_BC1	TG_BC1 obtained by crossing TG_BF_F1 transgenic females with BF_2 gen wild-type males	100
**Introgressed strain after six backcrosses**			
BC6	TG_BC6	Introgressed BC6 transgenic strain after six serial backcrosses	
	TG_ML_BC6	TG_BC6 obtained by crossing TG_ML_BC5 transgenic females with ML_7 gen wild-type males	100
	TG_BF_BC6	TG_BC6 obtained by crossing TG_BF_BC5 transgenic females with BF_7 gen wild-type males	100
**Total**			1400

Table S2. **Life table analysis** computed in three parental strains, Ag(PMB)1, Mali-NIH (ML) and BF_Ac(WT) (BF), the G3 strain from which the transgenic Ag(PMB)1 was derived, in the F1 and BC1 generations produced during introgression and in the introgressed transgenic strains after six backcrosses (ML_BC6 and BF_BC6). Egg number, hatching rate, larval mortality, total, male and female pupal mortality were reported (Mean ± Standard Error).

	Male ratio (Observed)	Egg number	Hatching	Larval Mortality	Pupal Mortality (Total)	Pupal Mortality (♀)	Pupal Mortality (♂)
**Parental strain (P)**							
Wild-type recipient strains							
1. ML (♀) x ML (♂)	0.535 ± 0.016	970 ± 164	0.517 ± 0.021	0.474 ± 0.049	0.153 ± 0.017	0.189 ± 0.026	0.114 ± 0.013
2. BF (♀) x BF (♂)	0.508 ± 0.008	3214 ± 647	0.799 ± 0.015	0.073 ± 0.023	0.057 ± 0.008	0.054 ± 0.009	0.060 ± 0.009
Transgenic donor strain							
Ag(PMB)1 (♀) x G3 (♂)	0.538 ± 0.008	7301 ± 1279	0.848 ± 0.024	0.075 ± 0.012	0.086 ± 0.012	0.088 ± 0.021	0.086 ± 0.006
G3 (♀) x Ag(PMB)1 (♂)	0.889 ± 0.013	5700 ± 1139	0.827 ± 0.025	0.067 ± 0.005	0.154 ± 0.012	0.158 ± 0.015	0.117 ± 0.041
Control							
G3 (♀) x G3 (♂)	0.508 ± 0.006	6966 ± 407	0.883 ± 0.015	0.120 ± 0.008	0.098 ± 0.012	0.122 ± 0.017	0.072 ± 0.009
**Experimental crosses (F1 and BC1)**							
1. Ag(PMB)1 (♀) x ML (♂)	0.503 ± 0.009	4118 ± 1073	0.496 ± 0.045	0.158 ± 0.017	0.037 ± 0.009	0.026 ± 0.004	0.035 ± 0.015
2. Ag(PMB)1 (♀) x BF (♂)	0.505 ± 0.014	6914 ± 704	0.829 ± 0.038	0.114 ± 0.019b	0.084 ± 0.009	0.114 ± 0.009	0.053 ± 0.014
1. TG_ML_F1 (♀) x ML (♂)	0.536 ± 0.101	4291 ± 849	0.740 ± 0.032	0.248 ± 0.050	0.076 ± 0.011	0.084 ± 0.015	0.068 ± 0.019
2. TG_BF_F1 (♀) x BF (♂)	0.533 ± 0.013	9249 ± 1181	0.895 ± 0.018	0.139 ± 0.019	0.077 ± 0.011	0.097 ± 0.017	0.055 ± 0.009
**Introgressed strain after six backcrosses (BC6)**							
1. TG_ML_BC6							
TG_ML_BC6 (♀) x ML (♂)	0.535 ± 0.012	4780 ± 492	0.684 ± 0.037	0.175 ± 0.035	0.080 ± 0.005	0.091 ± 0.011	0.068 ± 0.004
TG_ML_BC6 (♀) x NonTG_ML_BC6 (♂)	0.527 ± 0.011	7598 ± 1687	0.806 ± 0.038	0.118 ± 0.008	0.087 ± 0.016	0.098 ± 0.017	0.076 ± 0.017
ML (♀) x TG_ML_BC6 (♂)	0.989 ± 0.003	901 ± 146	0.531 ± 0.052	0.121 ± 0.027	0.093 ± 0.027	0.092 ± 0.026	0.167 ± 0.064
NonTG_ML_BC6 (♀) x TG_ML_BC6 (♂)	0.986 ± 0.003	2978 ± 786	0.769 ± 0.026	0.165 ± 0.025	0.081 ± 0.014	0.079 ± 0.014	0.096 ± 0.057
Control							
NonTG_ML_BC6 (♀) x NonTG_ML_BC6 (♂)	0.548 ± 0.009	3221 ± 425	0.715 ± 0.039	0.113 ± 0.020	0.160 ± 0.029	0.152 ± 0.031	0.171 ± 0.030

2. TG_BF_BC6							
TG_BF_BC6 (♀) x BF (♂)	0.493 ± 0.014	6085 ± 1367	0.743 ± 0.053	0.232 ± 0.068	0.051 ± 0.089	0.060 ± 0.009	0.038 ± 0.008
TG_BF_BC6 (♀) x NonTG_BF_BC6 (♂)	0.518 ± 0.012	6231 ± 1414	0.823 ± 0.013	0.145 ± 0.001	0.058 ± 0.006	0.052 ± 0.007	0.061 ± 0.012
BF (♀) x TG_BF_BC6 (♂)	0.983 ± 0.002	4208 ± 985	0.703 ± 0.038	0.289 ± 0.051	0.076 ± 0.009	0.076 ± 0.009	0.059 ± 0.041
NonTG_BF_BC6 (♀) x TG_BF_BC6 (♂)	0.990 ± 0.002	4708 ± 975	0.740 ± 0.017	0.283 ± 0.031	0.069 ± 0.008	0.068 ± 0.008	0.167 ± 0.008
Control							
NonTG_BF_BC6 (♀) x NonTG_BF_BC6 (♂)	0.509 ± 0.009	6281 ± 1037	0.747 ± 0.031	0.203 ± 0.072	0.098 ± 0.016	0.076 ± 0.008	0.122 ± 0.029

Table S3. 50% adult mortality on mean days and 95% confidence intervals were reported for adult survival in small cages for three parental strains, Ag(PMB)1, Mali-NIH (ML) and BF_Ac(WT) (BF), in the F1 and BC1 generations produced during introgression and in the introgressed transgenic strains after six backcrosses with ML (ML_BC6, 1 experiment) and BF (BF_BC6, 2 experiment).

	Adult Survival	♂ Adult Survival	♀ Adult Survival
**Parental strain (P)**			
Wild-type recipient strains			
1. ML (♀) x ML (♂)	17 [14-20]	17 [14-20]	17 [15-19]
2. BF (♀) x BF (♂)	19 [18-20]	19 [18-20]	19 [18-20]
Transgenic donor strain			
Ag(PMB)1 (♀) x G3 (♂)			
Experiment 1	15 [11-19]	15 [11-19]	14 [11-17]
Experiment 2	18 [17-19]	17 [14-20]	18 [17-20]
**Experimental crosses (F1 and BC1)**			
1. Ag(PMB)1 (♀) x ML (♂)	21 [15-25]	19 [14-24]	22 [15-29]
2. Ag(PMB)1 (♀) x BF (♂)	21 [20-21]	20 [19-21]	21 [20-21]
1. TG_ML_F1 (♀) x ML (♂)	17 [16-18]	18 [17-19]	17 [16-18]
2. TG_BF_F1 (♀) x BF (♂)	19 [18-20]	19 [15-23]	19 [18-20]
**Introgressed strain after six backcrosses (BC6)**			
1. TG_ML_BC6			
TG_ML_BC6 (♀) x ML (♂)	18 [15-21]	19 [17-21]	18 [15-21]
2. TG_BF_BC6			
TG_BF_BC6 (♀) x BF (♂)	17 [16-18]	16 [13-19]	18 [16-20]

Table S4. Statistics associated to kernel density plots of F_{ST} values for the wildtype recipient strain Mali-NIH at generation 1 (ML_1 gen) and BF_Ac(WT) at generation 1 (BF_1 gen) versus the transgenic donor strain Ag(PMB)1, transgenic F1 and BC1 generation strains produced during introgression process, the backcrossed transgenic BC6 progeny and three wildtype strains used as controls, (G3, paternal wildtype strain ML at generation 2 and 7).

	2R	2L	3R	3L	X		2R	2L	3R	3L	X
**Mean F_{ST}**	**ML Introgression**						**BF Introgression**				
ML_1 gen vs Ag(PMB)1	0.531	0.556	0.504	0.509	0.796	BF_1 gen vs Ag(PMB)1	0.360	0.416	0.352	0.366	0.536
ML_1 gen vs G3	0.529	0.527	0.509	0.515	0.818	BF_1 gen vs G3	0.356	0.388	0.357	0.369	0.553
ML_1 gen vs TG_ML_F1	0.244	0.285	0.224	0.228	0.326	BF_1 gen vs TG_BF_F1	0.142	0.175	0.124	0.127	0.180
ML_1 gen vs TG_ML_BC1	0.166	0.216	0.110	0.112	0.140	BF_1 gen vs TG_BF_BC1	0.105	0.173	0.068	0.066	0.090
ML_1 gen vs TG_ML_BC6	0.144	0.192	0.042	0.051	0.030	BF_1 gen vs TG_BF_BC6	0.091	0.166	0.053	0.049	0.099
ML_1 gen vs ML_2 gen	0.034	0.029	0.031	0.032	0.027	BF_1 gen vs BF_2 gen	0.044	0.046	0.050	0.047	0.090
ML_1 gen vs ML_7 gen	0.036	0.039	0.047	0.048	0.029	BF_1 gen vs BF_7 gen	0.051	0.054	0.057	0.053	0.104
**Standard Deviation F_{ST} (sd)**											
ML_1 gen vs Ag(PMB)1	0.185	0.165	0.207	0.214	0.212	BF_1 gen vs Ag(PMB)1	0.158	0.149	0.151	0.176	0.197
ML_1 gen vs G3	0.191	0.163	0.206	0.216	0.202	BF_1 gen vs G3	0.154	0.138	0.156	0.179	0.202
ML_1 gen vs TG_ML_F1	0.089	0.099	0.102	0.101	0.107	BF_1 gen vs TG_BF_F1	0.071	0.075	0.059	0.066	0.084
ML_1 gen vs TG_ML_BC1	0.068	0.080	0.059	0.053	0.051	BF_1 gen vs TG_BF_BC1	0.059	0.075	0.035	0.033	0.046
ML_1 gen vs TG_ML_BC6	0.075	0.082	0.025	0.028	0.029	BF_1 gen vs TG_BF_BC6	0.063	0.071	0.034	0.027	0.070
ML_1 gen vs ML_2 gen	0.024	0.021	0.023	0.022	0.025	BF_1 gen vs BF_2 gen	0.028	0.026	0.035	0.029	0.073
ML_1 gen vs ML_7 gen	0.027	0.030	0.042	0.051	0.028	BF_1 gen vs BF_7 gen	0.034	0.032	0.041	0.035	0.075
**Skewness (G1)**											
ML_1 gen vs Ag(PMB)1	0.325	-0.275	0.583	0.328	-0.847	BF_1 gen vs Ag(PMB)1	0.855	0.455	1.073	0.639	0.363
ML_1 gen vs G3	0.298	0.080	0.567	0.293	-1.043	BF_1 gen vs G3	0.899	0.741	1.069	0.673	0.221
ML_1 gen vs TG_ML_F1	0.281	-0.270	0.537	0.454	-0.489	BF_1 gen vs TG_BF_F1	1.253	0.735	1.459	1.142	0.814
ML_1 gen vs TG_ML_BC1	0.419	-0.136	0.645	0.514	0.108	BF_1 gen vs TG_BF_BC1	1.782	0.722	1.806	1.707	1.180
ML_1 gen vs TG_ML_BC6	0.825	0.270	1.286	1.172	1.943	BF_1 gen vs TG_BF_BC6	1.912	0.798	1.886	1.984	1.301
ML_1 gen vs ML_2 gen	1.639	1.473	1.909	2.028	1.536	BF_1 gen vs BF_2 gen	2.340	1.539	2.060	1.822	1.411
ML_1 gen vs ML_7 gen	1.869	1.520	3.446	2.558	1.773	BF_1 gen vs BF_7 gen	1.981	1.391	1.820	1.821	1.099
**G1 standard error (SES)**											

ML_1 gen vs Ag(PMB)1	0.011	0.012	0.012	0.013	0.018	BF_1 gen vs Ag(PMB)1	0.011	0.012	0.012	0.013	0.018
ML_1 gen vs G3	0.011	0.012	0.012	0.013	0.018	BF_1 gen vs G3	0.011	0.012	0.012	0.013	0.018
ML_1 gen vs TG_ML_F1	0.011	0.012	0.012	0.013	0.017	BF_1 gen vs TG_BF_F1	0.011	0.012	0.011	0.013	0.017
ML_1 gen vs TG_ML_BC1	0.011	0.012	0.012	0.013	0.017	BF_1 gen vs TG_BF_BC1	0.011	0.012	0.011	0.013	0.017
ML_1 gen vs TG_ML_BC6	0.011	0.012	0.013	0.014	0.028	BF_1 gen vs TG_BF_BC6	0.011	0.012	0.011	0.013	0.017
ML_1 gen vs ML_2 gen	0.014	0.016	0.014	0.016	0.029	BF_1 gen vs BF_2 gen	0.011	0.012	0.012	0.013	0.017
ML_1 gen vs ML_7 gen	0.014	0.016	0.014	0.016	0.029	BF_1 gen vs BF_7 gen	0.011	0.012	0.012	0.013	0.018
**Zg1 (teststat) = G1/SES [a]**											
ML_1 gen vs Ag(PMB)1	29.89 Pos	-22.80 Neg	49.4 Pos	24.46 Pos	-46.68 Neg	BF_1 gen vs Ag(PMB)1	79.13 Pos	38.12 Pos	91.95 Pos	48.09 Pos	20.22 Pos
ML_1 gen vs G3	27.54 Pos	6.70 Pos	48.41 Pos	21.96 Pos	-58.27 Neg	BF_1 gen vs G3	83.85 Pos	62.396 Pos	92.202 Pos	51.017 Pos	12.491 Pos
ML_1 gen vs TG_ML_F1	26.16 Pos	-22.62 Neg	46.27 Pos	34.38 Pos	-28.23 Neg	BF_1 gen vs TG_BF_F1	118.48 Pos	62.37 Pos	128.15 Pos	88.23 Pos	48.10 Pos
ML_1 gen vs TG_ML_BC1	38.93 Pos	-11.30 Neg	55.40 Pos	38.7 Pos	6.23 Pos	BF_1 gen vs TG_BF_BC1	168.54 Pos	61.24 Pos	158.80 Pos	131.98 Pos	69.99 Pos
ML_1 gen vs TG_ML_BC6	75.98 Pos	22.67 Pos	101.12 Pos	85.87 Pos	68.29 Pos	BF_1 gen vs TG_BF_BC6	180.85 Pos	67.75 Pos	165.71 Pos	153.50 Pos	75.29 Pos
ML_1 gen vs ML_2 gen	119.75 Pos	90.69 Pos	136.80 Pos	123.42 Pos	52.62 Pos	BF_1 gen vs BF_2 gen	220.44 Pos	129.39 Pos	177.81 Pos	139.46 Pos	81.11 Pos
ML_1 gen vs ML_7 gen	136.48 Pos	92.95 Pos	246.36 Pos	155.64 Pos	61.10 Pos	BF_1 gen vs BF_7 gen	183.27 Pos	114.52 Pos	153.82 Pos	135.84 Pos	61.67 Pos
**Kurtosis (G2)**											
ML_1 gen vs Ag(PMB)1	0.068	0.203	-0.284	-0.511	0.033	BF_1 gen vs Ag(PMB)1	0.581	0.359	1.026	0.340	-0.297
ML_1 gen vs G3	-0.078	0.365	-0.349	-0.489	0.484	BF_1 gen vs G3	0.790	1.116	0.947	0.363	-0.580
ML_1 gen vs TG_ML_F1	0.239	-0.241	-0.153	-0.115	0.240	BF_1 gen vs TG_BF_F1	2.637	1.062	2.883	2.377	1.152
ML_1 gen vs TG_ML_BC1	0.624	-0.104	0.502	-0.073	0.994	BF_1 gen vs TG_BF_BC1	5.556	0.879	5.723	6.341	3.171
ML_1 gen vs TG_ML_BC6	1.211	0.064	3.782	4.547	9.929	BF_1 gen vs TG_BF_BC6	5.904	1.396	6.303	6.897	1.933
ML_1 gen vs ML_2 gen	5.846	6.132	10.560	8.027	5.334	BF_1 gen vs BF_2 gen	10.897	4.242	6.302	4.734	1.962
ML_1 gen vs ML_7 gen	7.347	4.552	16.124	9.493	5.719	BF_1 gen vs BF_7 gen	6.363	2.857	4.456	4.664	1.223
**G2 standard error (SEK)**											

ML_1 gen vsAg(PMB)1	0.022	0.024	0.024	0.027	0.036	BF_1 gen vsAg(PMB)1	0.022	0.024	0.023	0.027	0.036
ML_1 gen vsG3	0.021	0.024	0.023	0.027	0.036	BF_1 gen vsG3	0.021	0.024	0.023	0.026	0.035
ML_1 gen vs TG_ML_F1	0.022	0.024	0.023	0.026	0.035	BF_1 gen vs TG_BF_F1	0.021	0.024	0.023	0.026	0.034
ML_1 gen vs TG_ML_BC1	0.022	0.024	0.024	0.027	0.035	BF_1 gen vs TG_BF_BC1	0.021	0.024	0.023	0.026	0.035
ML_1 gen vs TG_ML_BC6	0.022	0.024	0.025	0.027	0.057	BF_1 gen vs TG_BF_BC6	0.021	0.024	0.023	0.026	0.034
ML_1 gen vs ML_2 gen	0.027	0.032	0.028	0.033	0.058	BF_1 gen vs BF_2 gen	0.021	0.024	0.023	0.026	0.035
ML_1 gen vs ML_7 gen	0.027	0.033	0.028	0.033	0.058	BF_1 gen vs BF_7 gen	0.022	0.024	0.024	0.027	0.036
**Zg2 (teststat) = G2/SEK [b]**											
ML_1 gen vsAg(PMB)1	3.13	8.44	-12.07	-19.02	0.94	BF_1 gen vsAg(PMB)1	26.91	15.03	43.95	12.82	-8.28
	Lepto	Lepto	Platy	Platy	Meso		Lepto	Lepto	Lepto	Lepto	Platy
ML_1 gen vsG3	-3.63	15.22	-14.91	-18.34	13.5	BF_1 gen vsG3	36.86	46.98	40.85	13.74	-16.42
	Platy	Lepto	Platy	Platy	Lepto		Lepto	Lepto	Lepto	Lepto	Platy
ML_1 gen vs TG_ML_F1	11.11	-10.08	-6.57	-4.34	6.92	BF_1 gen vs TG_BF_F1	124.65	45.05	126.57	91.83	34.04
	Lepto	Platy	Platy	Platy	Lepto		Lepto	Lepto	Lepto	Lepto	Lepto
ML_1 gen vs TG_ML_BC1	28.93	-4.34	21.57	-2.77	28.72	BF_1 gen vs TG_BF_BC1	262.71	37.30	251.61	245.14	94.01
	Lepto	Platy	Lepto	Platy	Lepto		Lepto	Lepto	Lepto	Lepto	Lepto
ML_1 gen vs TG_ML_BC6	55.77	2.67	148.73	166.5	174.51	BF_1 gen vs TG_BF_BC6	279.19	59.26	276.97	266.80	55.95
	Lepto	Lepto	Lepto	Lepto	Lepto		Lepto	Lepto	Lepto	Lepto	Lepto
ML_1 gen vs ML_2 gen	213.46	188.84	378.36	244.28	91.35	BF_1 gen vs BF_2 gen	513.23	178.36	272.01	181.23	56.41
	Lepto	Lepto	Lepto	Lepto	Lepto		Lepto	Lepto	Lepto	Lepto	Lepto
ML_1 gen vs ML_7 gen	268.20	139.19	576.303	288.7	98.57	BF_1 gen vs BF_7 gen	294.32	117.63	188.36	174.02	34.31
	Lepto	Lepto	Lepto	Lepto	Lepto		Lepto	Lepto	Lepto	Lepto	Lepto

[a] Pos = Positively skewed function; Neg = Negatively skewed function.
[b] Lepto = Leptokurtic function; Platy = Platykurtic function; Meso = Mesokurtic function

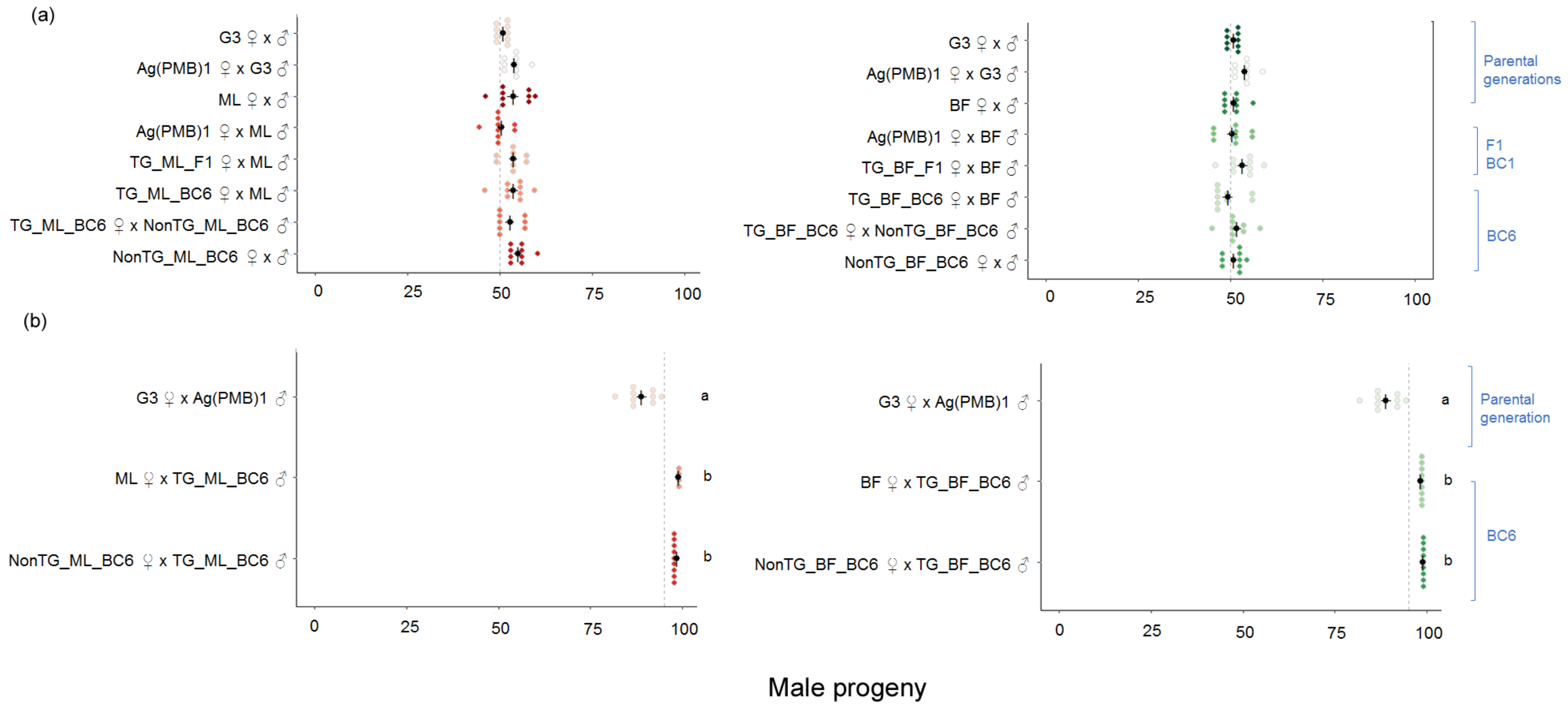

Figure S1. **The sex ratio** recorded in the *An. coluzzii* wild-type recipient strain Mali-NIH (red scale) and BF_Ac(WT) (green scale), the transgenic donor strain Ag(PMB)1, the G3 strain from which the transgenic line was derived, during introgression (F1 and BC1) and in the progenies obtained after six serial backcrossing, ML_BC6 and BF_BC6, using **a)** transgenic females and **b)** transgenic males. Dashed gray line indicates the expected male progeny: ~ 50% from cross Ag(PMB)1 ♀ x G3 ♂ and ~ 95% from cross G3 ♀ x Ag(PMB)1 ♂. Mean values showing different letters were significantly different at $P < 0.05$ according to the post-hoc non-parametric multiple Dunn test after providing significance within the generalized linear model (GLM). TG = transgenic mosquitoes, NonTG = non transgenic mosquitoes, Mali-NIH = ML and BF_Ac(WT) = BF

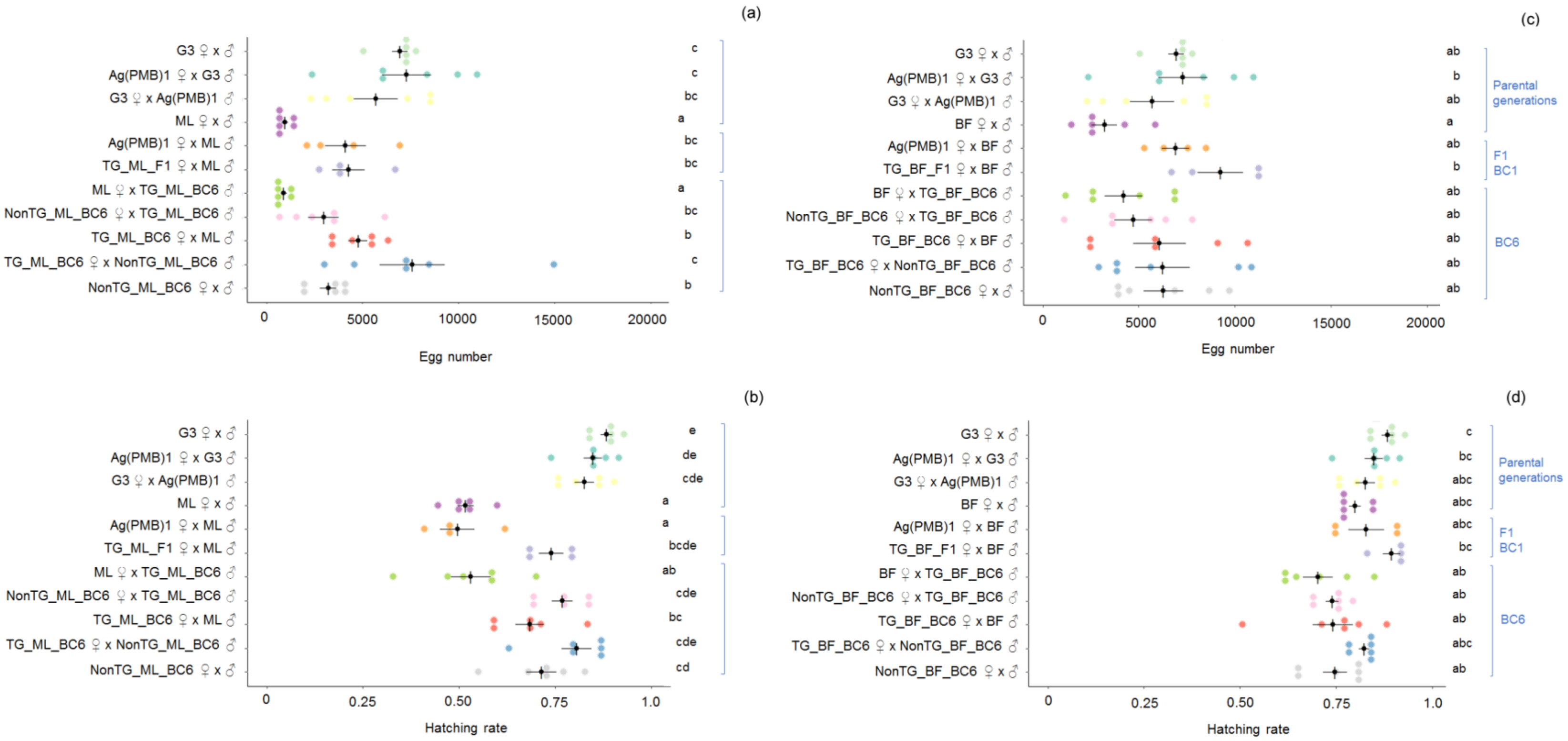

Figure S2. **Female fertility in terms of number of eggs and hatching rate** recorded in the transgenic donor strain Ag(PMB)1, the G3 strain from which the transgenic line was derived, during introgression (F1 and BC1) and in the BC6 backcrossed progenies, ML_BC6 and BF_BC6, introgressed using the *An. coluzzii* wild-type genetic background Mali-NIH (a-b) and BF_Ac(WT) (c-d). Mean values showing different letters were significantly different at P < 0. 05 according to the post-hoc non-parametric multiple Dunn test after providing significance within GLM. TG = transgenic mosquitoes, NonTG = non transgenic mosquitoes, Mali-NIH = ML and BF_Ac(WT) = BF

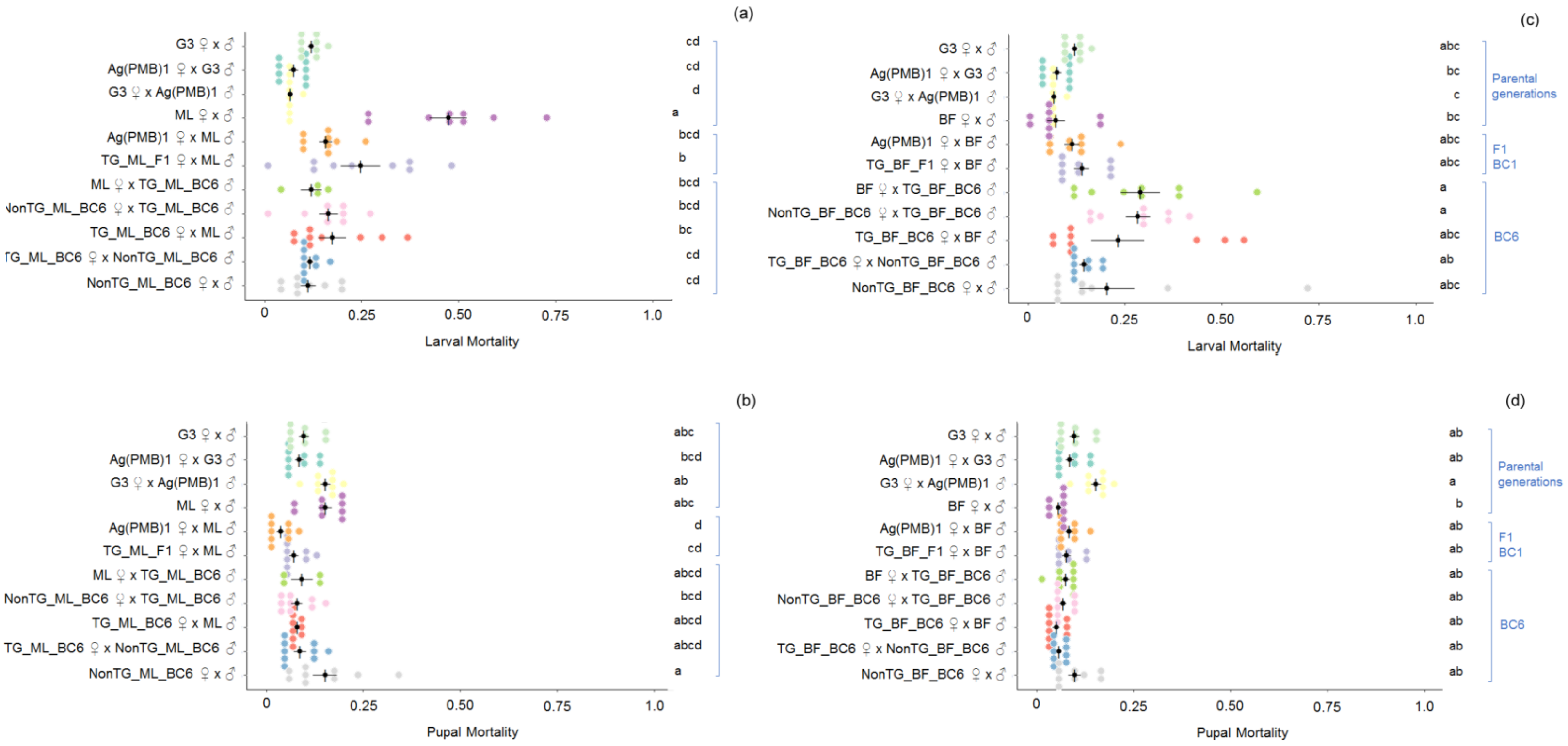

Figure S3. Larval mortality and pupal mortality recorded in the transgenic donor strain Ag(PMB)1, the G3 strain from which the transgenic line was derived, during introgression (F1 and BC1) and in the BC6 backcrossed progenies, ML_BC6 and BF_BC6, introgressed using the *An. coluzzii* wild-type genetic background Mali-NIH (a-b) and BF_Ac(WT) (c-d). Mean values showing different letters were significantly different at $P < 0.05$ according to the post-hoc non-parametric multiple Dunn test after providing significance within GLM. TG = transgenic mosquitoes, NonTG = non transgenic mosquitoes, Mali-NIH = ML and BF_Ac(WT) = BF

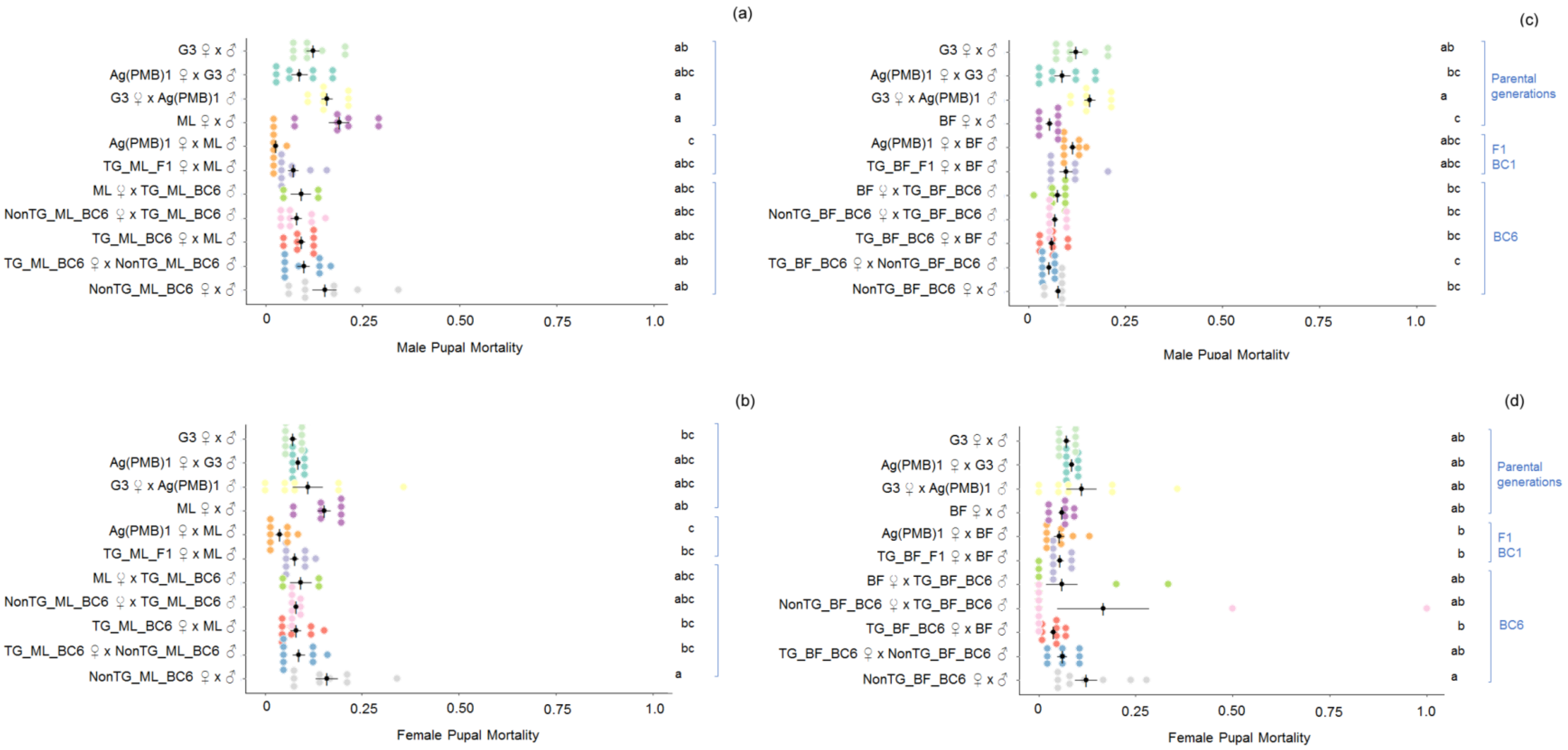

Figure S4. **Male and female pupal mortality** recorded in the transgenic donor strain Ag(PMB)1, the G3 strain from which the transgenic line was derived, during introgression (F1 and BC1) and in the BC6 backcrossed progenies, ML_BC6 and BF_BC6, introgressed using the *An. coluzzii* wild-type genetic background Mali-NIH (a-b) and BF_Ac(WT) (c-d). Mean values showing different letters were significantly different at P < 0. 05 according to the post-hoc non-parametric multiple Dunn test after providing significance within GLM. TG = transgenic mosquitoes, NonTG = non transgenic mosquitoes, Mali-NIH = ML and BF_Ac(WT) = BF.

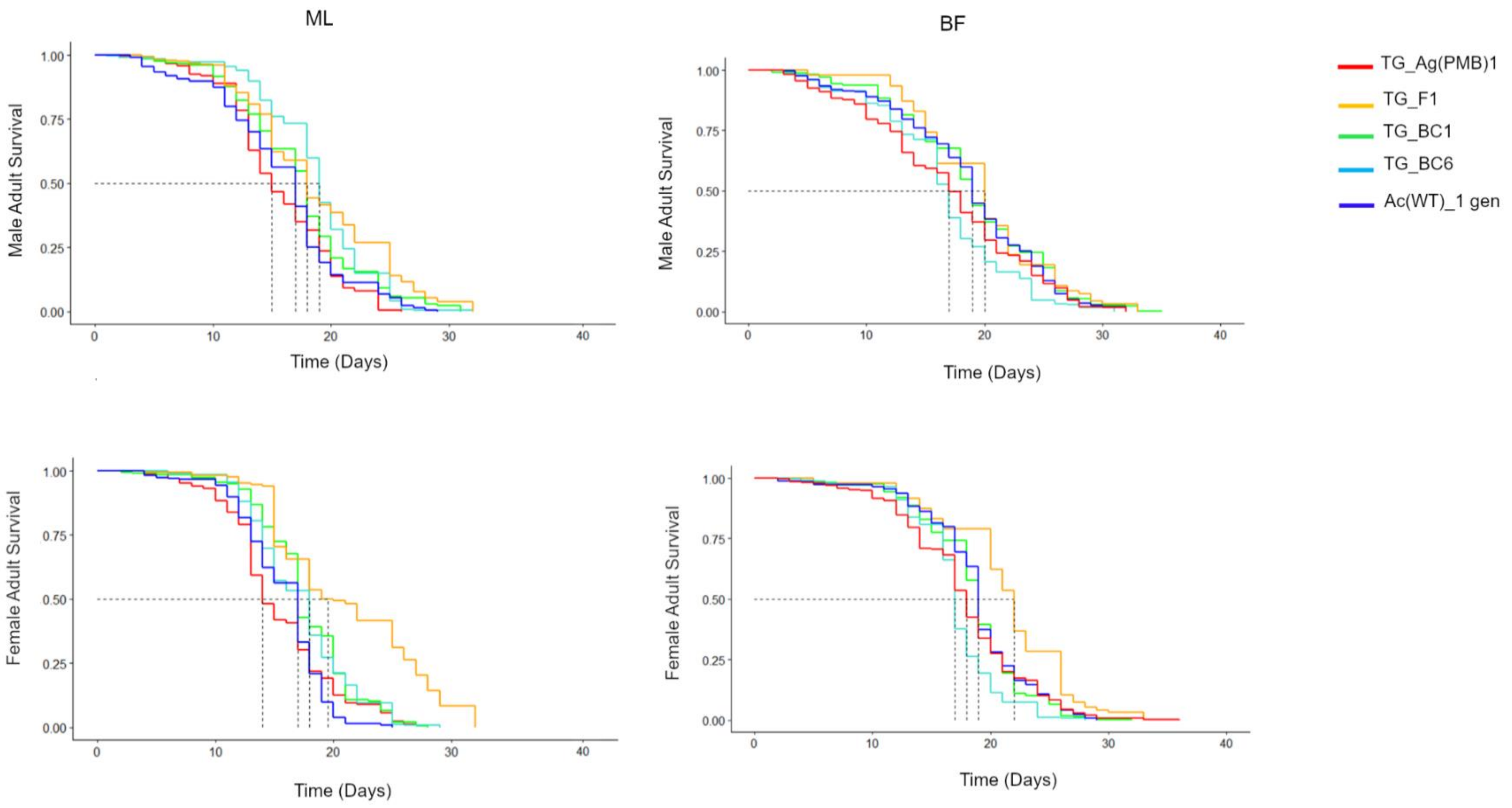

Figure S5. Adult survival for male and female adult mosquitoes in small cages (Time = Days) estimated for the donor transgenic strain Ag(PMB)1, generations F1 and BC1, and in the BC6 transgenic progenies backcrossed using the wild-type genetic background Mali-NIH (ML) and BF_Ac(WT) (BF). TG = transgenic mosquitoes.

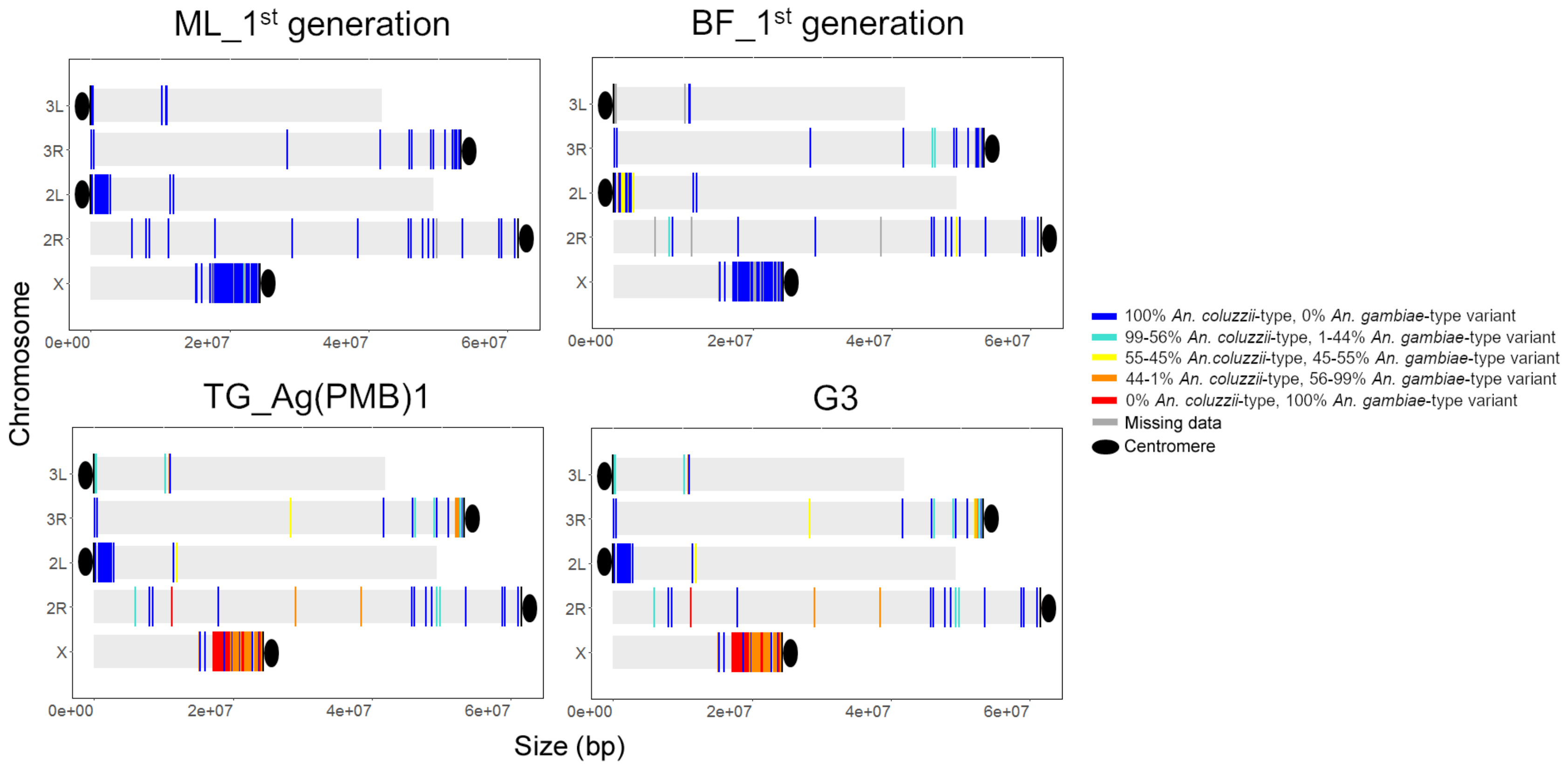

Figure S6. **Patterns of genetic admixture between *An. gambiae* and *An. coluzzii*** along X, 2R, 2L, 3R and 3L chromosomes for three parental strains (Ag(PMB)1, Mali-NIH and BF_Ac(WT)) and G3 control using ancestry informative markers (AIMs). First mosquito generation used for starting introgression process was displayed for each strain. Vertical coloured bars represent 329 informative SNPs for *An. coluzzi* and *An. gambiae* located in the chromosome X (236 loci), 2R (21 loci), 2L (38 loci), 3R (24 loci) and 3L (10 loci). The frequencies of SNP variants were computed for 100 individual mosquitoes for each strain by Pool-Seq approach. Chromosomal position of each SNP frequency-type is plotted on the x-axis (size in bp). TG = transgenic mosquitoes.

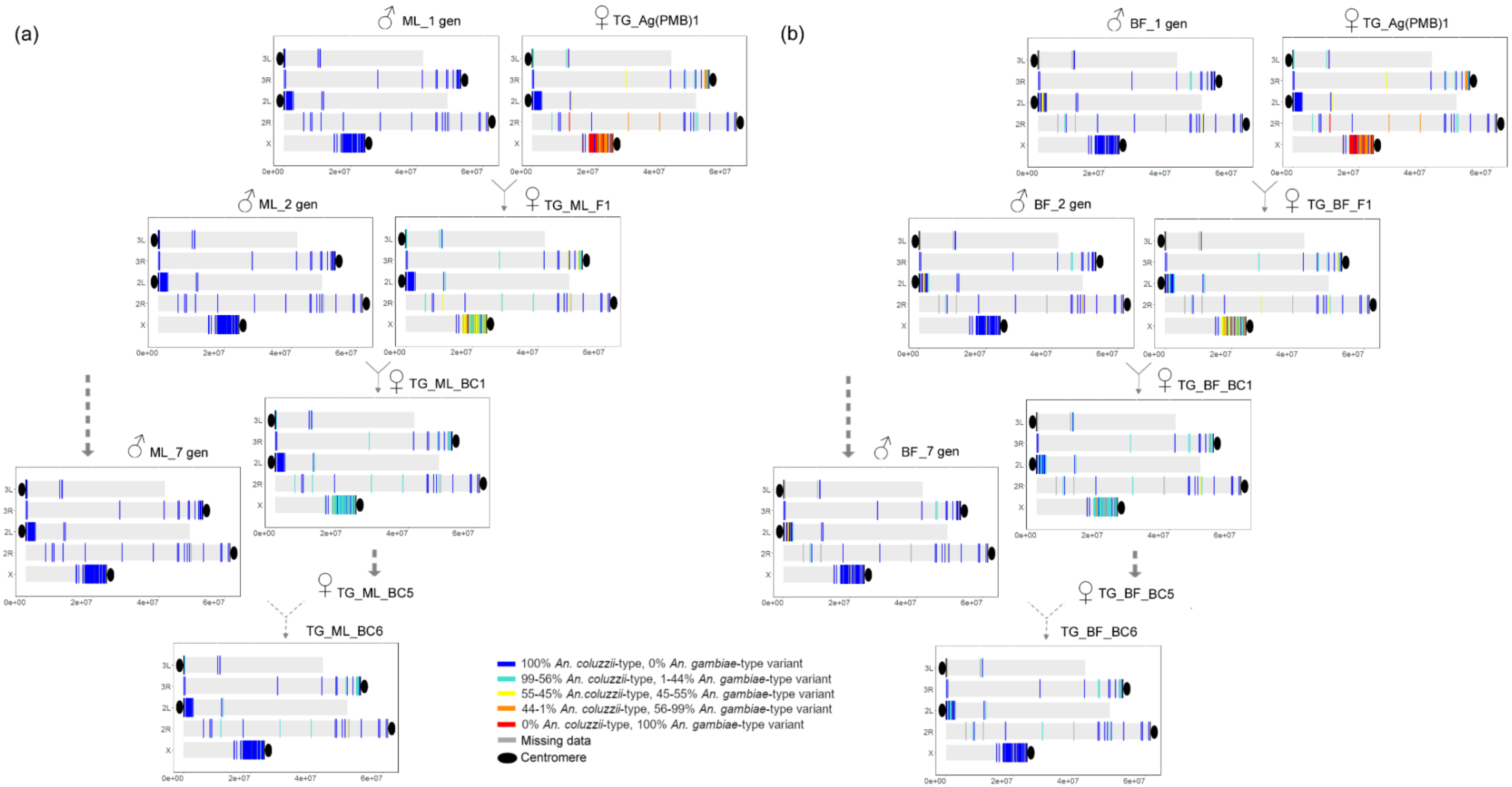

Figure S7. **Following the serial introgression of the sex ratio distortion transgene (TG)** from Ag(PMB)1 into two genetic backgrounds, ML (a) and BF (b) by ancestry informative markers (AIMs). Vertical coloured bars represent 329 informative SNPs for *An. coluzzi* and *An. gambiae* located in the chromosome X (236 loci), 2R (21 loci), 2L (38 loci), 3R (24 loci) and 3L (10 loci). The frequencies of SNPs were computed for 100 individual mosquitoes for each strain by Pool-Seq approach. Chromosomal position of each SNP frequency-type is plotted on the x-axis (size in bp). TG = transgenic mosquitoes.

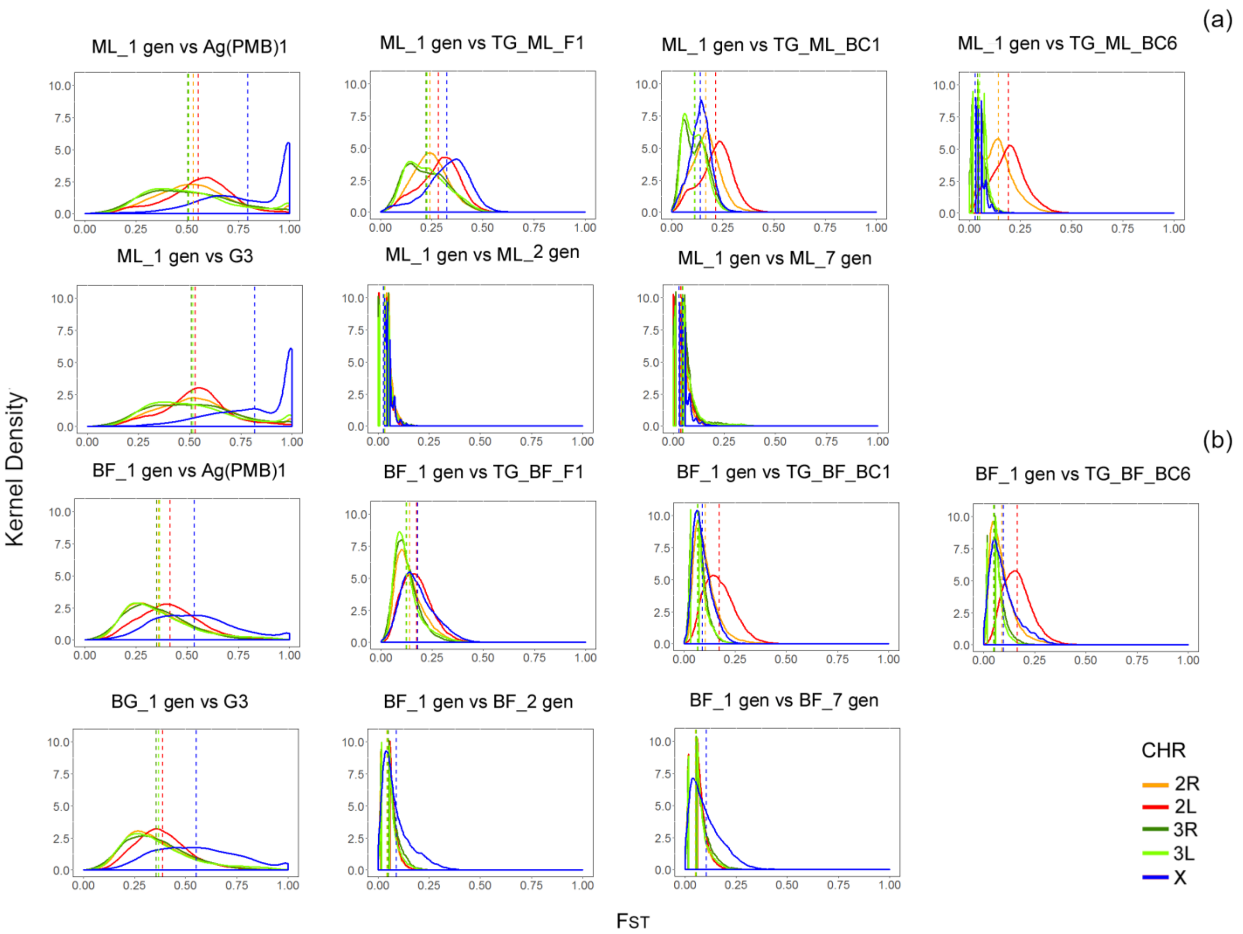

Figure S8. Kernel density plots of F_{ST} for parental and introgressed transgenic mosquito strains computed for each chromosome arm. Kernel density plots of genetic differentiation F_{ST} values for the wildtype recipient strain Mali-NIH at generation 1 (ML_1 gen) (a) and BF_Ac(WT) at generation 1 (BF_1 gen) (b) versus the transgenic donor strain Ag(PMB)1, transgenic F1 and BC1 offspring produced during backcrossing process, the backcrossed transgenic BC6 progeny and three wildtype strains used as controls, (G3, recipient wildtype strain at generation 2 and 7). The distributions of F_{ST} were calculated over 1 kb sliding windows for each chromosome arm. The median value of each kernel density distribution is reported as dotted line.

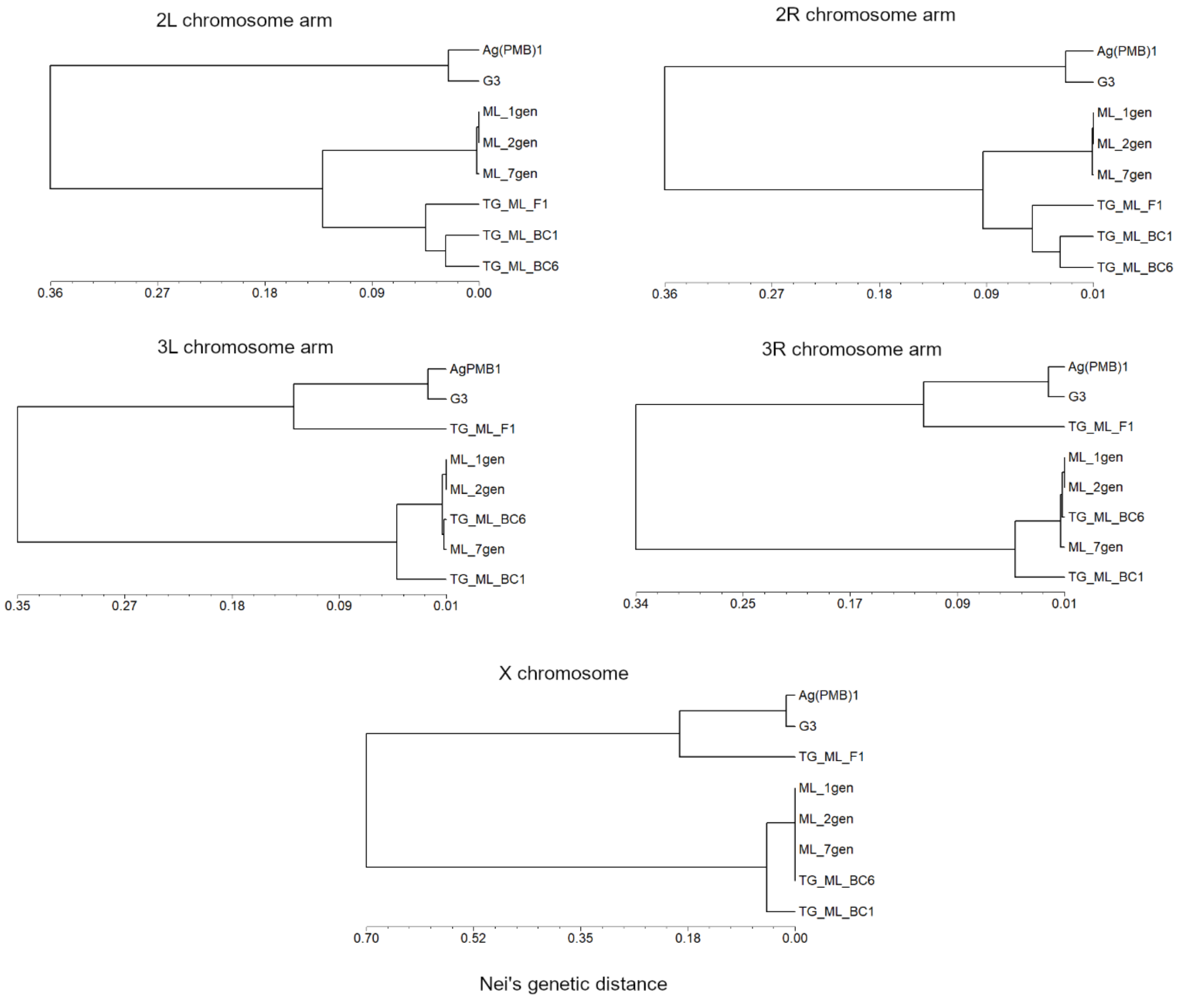

Figure S9. **Clustering tree analysis for ML introgression** based on the computation of the pairwise Nei's genetic distances between the wildtype recipient strain ML at 1, 2, and 7 generation, the transgenic donor strain Ag(PMB)1, the G3 strain from which the transgenic line was derived, transgenic F1 and BC1 generation strains produced during introgression process, and the backcrossed transgenic BC6 progeny using 1,295,912 SNPs (2L chromosome arm), 1,287,772 SNPs (2R chromosome arm), 882,658 SNPs (3L chromosome arm), 1,256,628 SNPs (3R chromosome arm) and 227,335 SNPs (X chromosome). All nodes are supported by 100% of 100 bootstrap replicates.

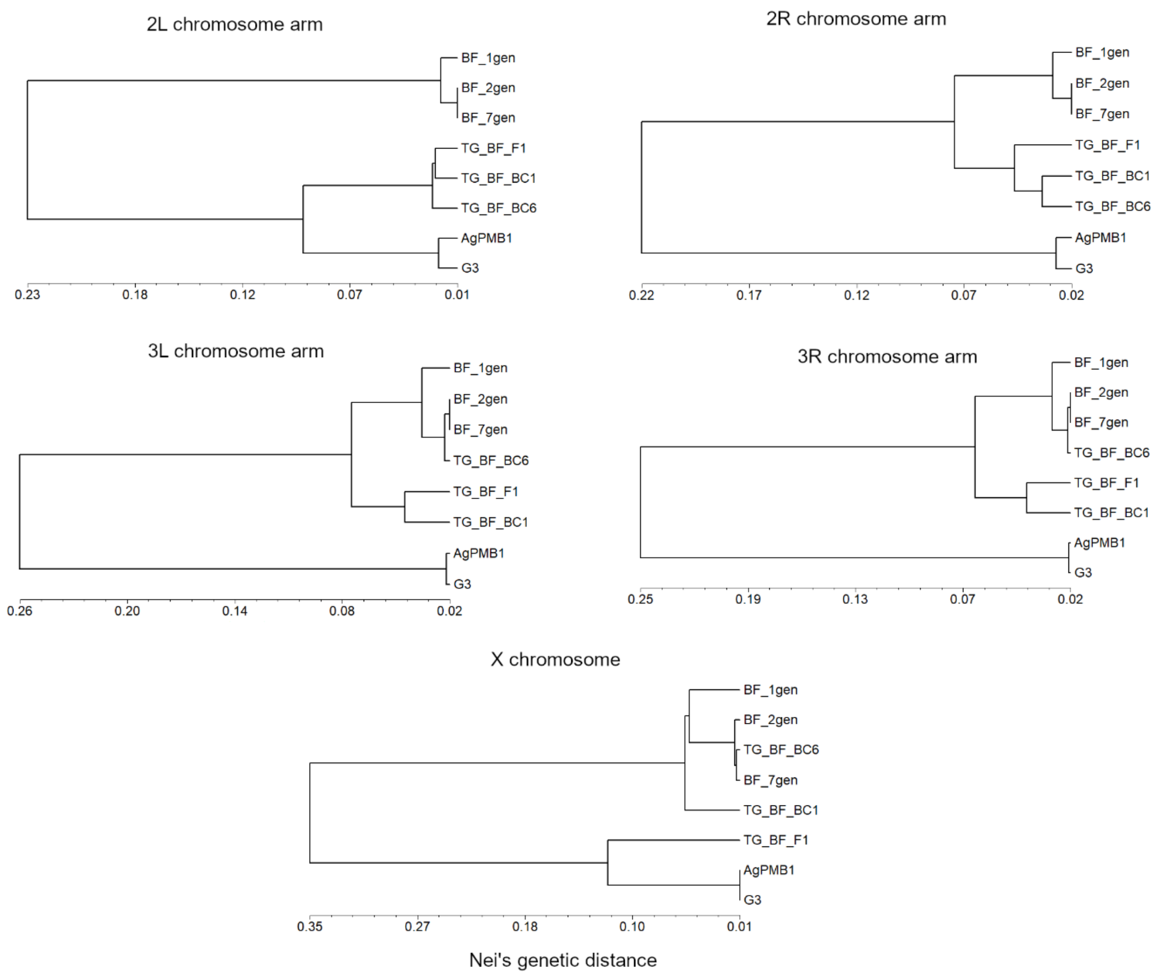

Figure S10. **Clustering tree analysis for BF introgression** based on the computation of the pairwise Nei's genetic distances between the wildtype recipient strain BF at 1, 2, and 7 generation (BF), the transgenic donor strain Ag(PMB)1, the G3 strain from which the transgenic line was derived, transgenic F1 and BC1 generation strains produced during introgression process, and the backcrossed transgenic BC6 progeny using 1,355,120 SNPs (2L chromosome arm), 1,282,942 SNPs (2R chromosome arm), 896,629 SNPs (3L chromosome arm), 1,288,667 SNPs (3R chromosome arm) and 260,030 SNPs (X chromosome). All nodes are supported by 100% of 100 bootstrap replicates.

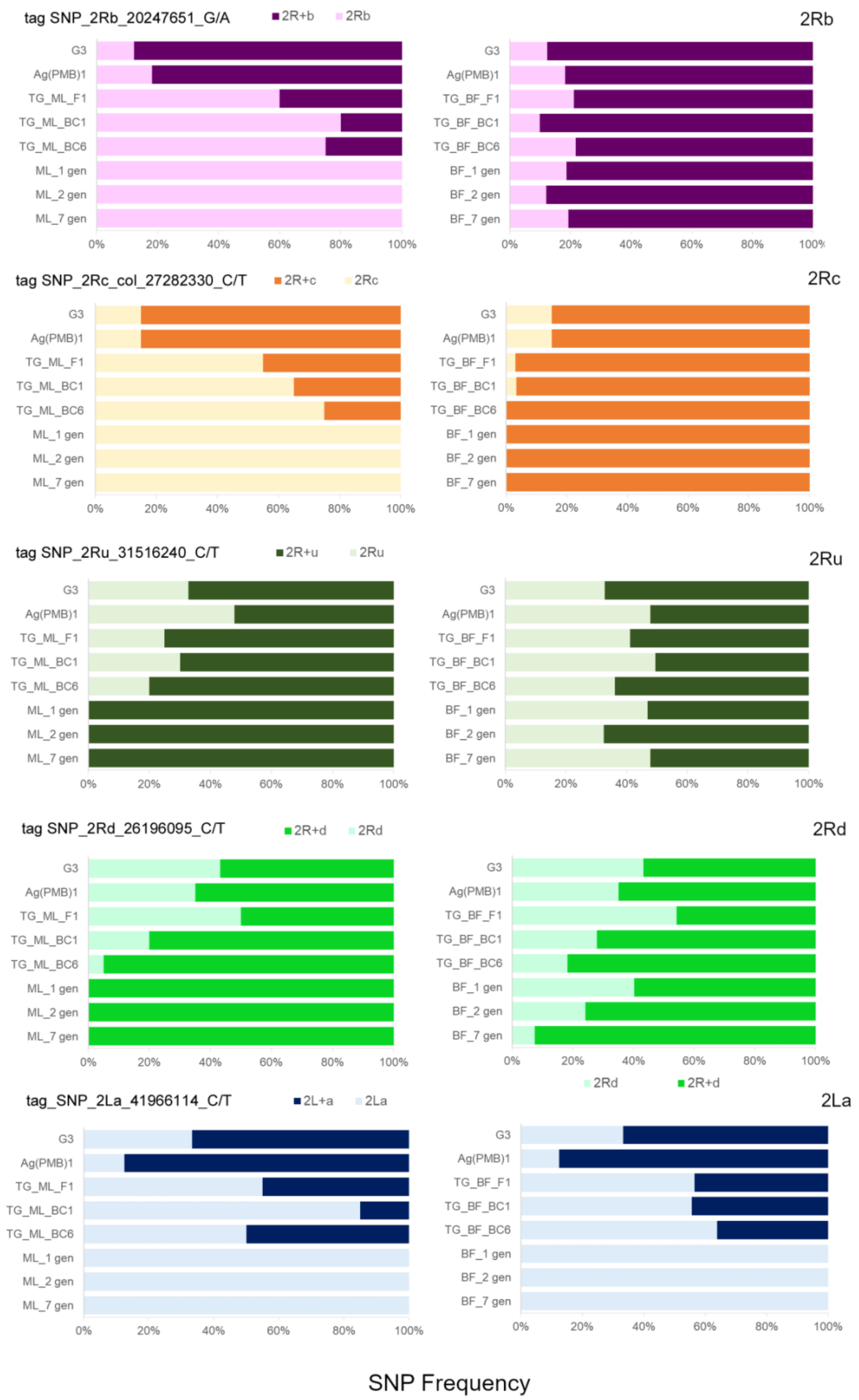

Figure S11. Frequency variation of tag SNPs associated with five chromosome inversions (2Rb, 2Rc, 2Ru, 2Rd and 2La) in *An. gambiae* and *An. coluzzii* assayed during the serial backcrossing of the transgene from Ag(PMB)1 into two genetic backgrounds, ML and BF.

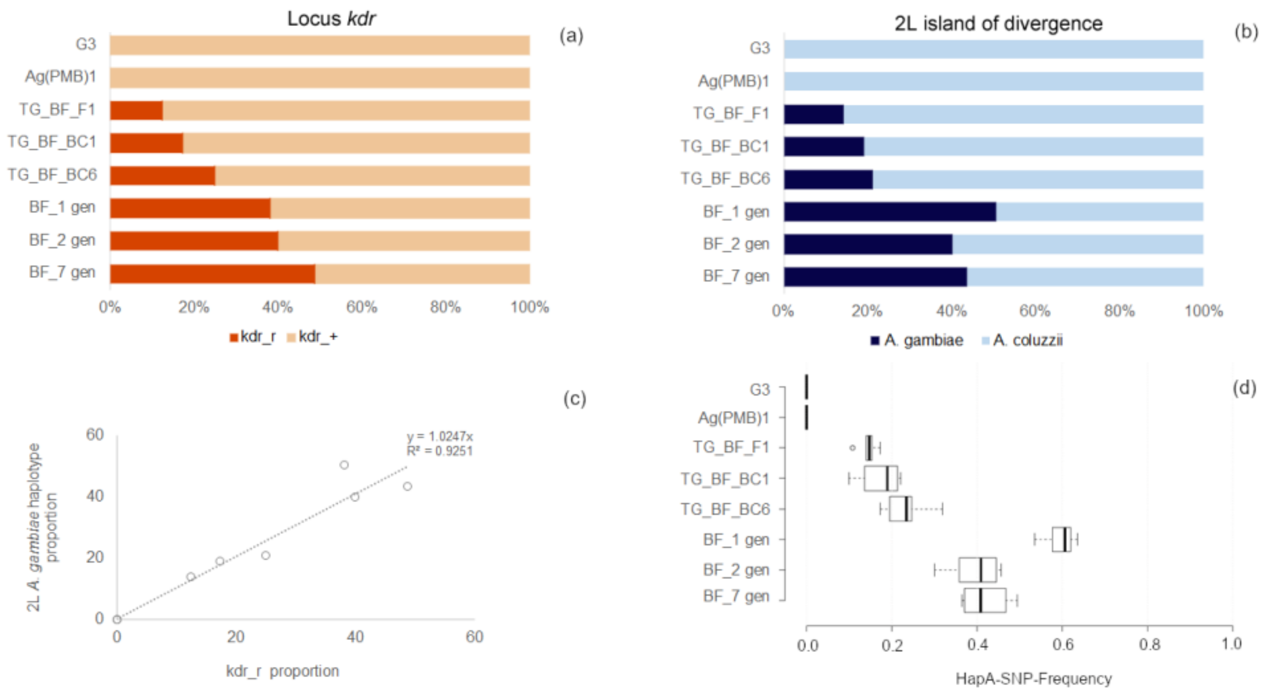

Figure S12. Distribution of the kdr_r allele, *An. gambiae* species-specific haplotype and HapA-tagging SNPs in the 2L divergence island during the serial backcrossing of transgene from Ag(PMB)1 to BF_Ac(WT). Frequency proportion (%) of the L1014F SNP variants (*kdr* resistance) (a), 2L divergence island species-specific haplotypes (b) computed by Pool-Seq approach and DIS Assay respectively, in the transgenic donor strain Ag(PMB)1, G3 control, wildtype recipient strain BF_Ac(WT) at generation 1, 2 and 7 (BF_1 gen, BF_2, gen BF_7 gen), transgenic F1 and BC1 generation strains produced during introgression process, and the backcrossed transgenic BC6 progeny. (c) The regression analysis between *kdr_r* resistance allele and 2L *An. gambiae* haplotype proportion and (d) the frequencies of five HapA-tagging markers for susceptibility to *P. falciparum* are also reported.

Whole genome pooled sequencing

After genomic DNA quantification by Qubit fluorometer (dsDNA, BR, Invitrogen), equal amounts of DNA from 100 individuals per group were pooled, resulting in a pool size of 200 ng RNA-free genomic DNA per population. Eight (Ag(PMB)1, G3, ML at generation 1, 2 and 7, TG_ML_F1, TG_ML_BC1 and TG_ML_BC6) and six (BF at generation 1, 2 and 7, TG_BF_F1, TG_BF_BC1 and TG_BF_BC6) libraries were separately prepared using the TrueSeq DNA nano kit and TruSeq dual indexing barcodes (Illumina), and sequenced to an expected mean coverage of 30X using Illumina NextSeq550 platform with paired-end, 150-bp reads. DNA libraries were prepared and sequenced at Polo d'Innovazione di Genomica, Genetica e Biologia Società Consortile R.L., Siena, Italy.

Demultiplexed 150bp paired-end reads were processed with the program Trimmomatic v0.36 (Bolger et al., 2014) to remove adapter sequences, to discard low-quality reads (Phred quality score = 20) and to be trimmed to a minimum length of 50bp. Trimmed paired-end reads were mapped to the PEST AgamP4.12 reference genome of *An. gambiae* available on Vectorbase (https://legacy.vectorbase.org/organisms/pest/agamp412) using Burrows-Wheeler Aligner tool *bwa mem* (v07-10-r789, Li & Durbin, 2009) by specifying the option –k 30 to meet the specificity of Pool-Seq data, increasing mapping accuracy with this semi-global mapping approach. The SAM alignment files were then converted to binary BAM files; furthermore, they were sorted and indexed using the software package SAMtools v1.10 (Li et al., 2009). The tool MarkDuplicates of the software package Picard (v.1.119 available at http://picard.sourceforge.net) was applied to mark and remove the optical duplicates from the alignments. The alignments were further filtered based on their mapping quality. Aiming at very

good mapping quality, reads with a quality score <20, singletons and unproper pairs were removed from the alignments using SAMtools. In order to perform a pairwise comparison of each pool vs the recipient wild-type strain, single nucleotide polymorphisms were called with SAMtools *mpileup* (Li et al., 2009). Each mpileup file was synchronized and again filtered for base quality (Q20) using the perl script *mpileup2sync.pl* of POPOOLATION2 (Kofler et al., 2011). The synchronized (sync) file is the input for POPOOLATION2 program and it contains all the information about all the SNPs detected and the respective allele frequencies. We implemented a number of stringent criteria to sort alleles for the subsequent analysis. The coverage along the genome and the coverage per quantile were calculated using the SAMtools *depth* and R *quantile* functions, respectively. In particular, we detected the coverage values at 1% increments and considered the 98% as maximum coverage value for each pool. In order to accurately estimate allele frequencies and correct for potential errors from copy number variations and mis-mappings, the minimum coverage of 20 and maximum coverage of 80 were used as thresholds for SNP identification in the first dataset comparison (ML at generation 1 vs Ag(PMB)1, G3, ML at generation 2 and 7, ML_TG_F1, ML_TG_BC1 and ML_TG_BC6). We set up 20 and 100 as minimum and maximum read coverage for the subsequent dataset comparison (BF at generation 1 vs Ag(PMB)1, G3, BF at generation 2 and 7, BF_TG_F1, BF_TG_BC1 and BF_TG_BC6). Thus, mpileup files were inspected for indels using the *identify-genomic-indel-regions.pl* script in POPOOLATION2 requiring a minimum count of two for an indel and indel-windows 5. The regions containing these indels were then detected and filtered out from each sync file using *filtersync-by-gtf.pl* script in the POPOOLATION2 suite. To allow for an unbiased comparability across data sets, the synchronized files were subsampled and normalized to a target coverage of 20 in order to synchronize coverage variations. The

POPOOLATION2 script *subsample-synchronized.pl* was also used to select all the SNPs with maximum coverage of 80 or 100 using the max-coverage option. The method parameter "withoutreplace" was applied to the normalization process.

The genetic differentiation index (F_{ST}) was then calculated using the scripts *fst-sliding.pl* in POPOOLATION2, using a sliding-window approach with a window size of 1000 bp and step size 1000 bp. Within each sliding window, at least 50% of SNPs had to fulfil the coverage threshold specified above. Pool size per population was set to 200 because 100 diploid mosquitoes were represented in each pool. The non-informative sites were removed from the analysis (--suppress-noninformative). Finally, the F_{ST} values were displayed along each chromosome as Manhattan plots using *qqman* R package (Turner, 2018). In addition, we used the R package LOKERN (https://cran.r-project.org/web/packages/ lokern/index.html) with a kernel regression smoothing algorithm to display smoothed F_{ST} values along each chromosome. Kernel density plots and their associated mean, median, skewness and kurtosis parameters were computed to evaluate the variation in density distributions of F_{ST} values on each chromosome during the introgression process using in house-R script.

Finally, Nei's genetic distances were computed between pool-samples using the *gendist* module implemented in PHYLIP v.3.68 (Felsenstein, 2005), from genome-wide allele frequencies obtained from POPOOLATION2 data processing. A PHYLIP input file was produced for each chromosome arm (2L, 2R, 3L, 3R, X) in each introgression experiment modifying the output *rc* files through in-house bash and R scripts. Following the computation of Nei's genetic distances between pools, a UPGMA (Unweighted Pair Group Method with Arithmetic mean) tree per chromosome arm was constructed. Bootstrap support for each tree was determined by resampling loci 100 times using SEQBOOT and summarized by CONSENSE

within PHYLIP. In addition, a supplemental set of 329 informative Ancestry Informative Markers (AIMs) located in the chromosome X (236 SNPs), 2R (21 SNPs), 2L (38 SNPs), 3R (24 SNPs) and 3L (10 SNPs) were screened in our pool datasets. We build up the *mpileup* and the synchronized files, inclusive of all the eight pools per introgression experiment 1 and 2 (Table S1). The allele frequency distribution for each AIM was estimated across pools with function *snp-frequency-diff.pl* implemented in POPOOLATION2 (*rc* files).

References

Bolger, A. M., Lohse, M., & Usadel, B. (2014). Trimmomatic: a flexible trimmer for Illumina sequence data. *Bioinformatics*, *30*(15), 2114–2120.https://doi.org/10.1093/bioinformatics/btu170

Felsenstein J. (2005) PHYLIP (phylogeny inference package). Department of Genome Sciences, University of Washington, Seattle.

Kofler, R., Pandey, R. V., & Schlötterer, C. (2011). PoPoolation2: identifying differentiation between populations using sequencing of pooled DNA samples (Pool-Seq). *Bioinformatics*, *27*(24), 3435–3436. https://doi.org/10.1093/bioinformatics/btr589.

Li, H., & Durbin, R. (2009). Fast and accurate short read alignment with Burrows-Wheeler transform. *Bioinformatics*, *25*(14), 1754–1760. https://doi.org/10.1093/bioinformatics/btp324.

Li, H., Handsaker, B., Wysoker, A., Fennell, T., Ruan, J., Homer, N., Marth, G., Abecasis, G., Durbin, R., & 1000 Genome Project Data Processing Subgroup (2009). The Sequence Alignment/Map format and SAMtools. *Bioinformatics*, *25*(16), 2078–2079. https://doi.org/10.1093/bioinformatics/btp352.

Turner, S.D. (2018). qqman: an R package for visualizing GWAS results using Q-Q and manhattan plots. *Journal of Open Source Software*, *3*(25), 731. https://doi.org/10.21105/joss.00731.
